# Multilevel onsite training and mentorship model to accelerate early childhood cancer diagnosis in Northwest Ethiopia: A quasi-experimental mixed method study

**DOI:** 10.1371/journal.pmed.1005132

**Published:** 2026-06-29

**Authors:** Mulugeta Ayalew Yimer, Alemayehu Teklu Toni, Nahom Worku Teshager, Degalem Tilahun Worku, Mulat Asrade Alemayehu, Yalew Melkamu Molla, Zewudu Andualem, Asefa Adimasu Tadesse

**Affiliations:** 1 Department of Pediatrics and Child Health, School of Medicine, College of Medicine and Health Sciences, University of Gondar, Gondar, Ethiopia; 2 Department of Environmental and Occupational Health and Safety, Institute of Public Health, College of Medicine and Health Sciences, University of Gondar, Gondar, Ethiopia; 3 Department of Epidemiology and Biostatistics, Institute of Public Health, College of Medicine and Health Sciences, University of Gondar, Gondar, Ethiopia; Makerere University Medical School, UGANDA

## Abstract

**Background:**

Childhood cancer survival in low-income countries remains below 20%, with late recognition and delayed referral commonly reported. In Northwest Ethiopia, care providers often lack the knowledge, skills, and support systems for recognizing early warning signs of pediatric malignancies. Community caregivers often present only after symptoms become advanced. The University of Gondar Comprehensive Specialized Hospital piloted a quality improvement initiative aimed at achieving earlier diagnoses. This study reports whether a multilevel onsite training and mentorship model could improve early recognition, referral practices, and timely diagnosis of childhood cancer in Northwest Ethiopia.

**Methods and findings:**

From January 2024 through September 2024, a quasi-experimental pre-post, mixed-methods study design was employed across three tiers of care: primary (e.g., health centers), secondary (general hospitals), and tertiary (specialized hospitals). Eighteen primary-level and 29 secondary-level clinicians completed intensive, on-site training (Ten and Seven days, respectively), while one thousand twenty health extension workers received pictorial outreach modules. A 6-month mentorship program combined monthly onsite visits with remote supervision. Mentees included general practitioners, nurses, health officers, and health extension workers mentored by a multidisciplinary team that included, for example, pediatric haemato-oncologists. Knowledge, attitude, and practice surveys and clinical chart reviews were conducted at baseline and 6 months post-implementation to evaluate patient-journey intervals. These quantitative assessments were integrated with qualitative interviews and focus groups grounded in the consolidated framework for implementation research. The intervention utilized specific curricula focusing on clinical recognition, referral protocols, and documentation.

In this study, the participants had a 100% response rate. Median knowledge scores at primary and secondary levels rose from 54.6 (95% CI: 36.4, 63.6) to 90.90 (95% CI: 81.8, 100.0) and from 36.36 (95% CI: 27.3, 45.5) to 90.91 (95% CI: 61.4, 93.7), respectively. Median practice proficiency increased 87.5 (95% CI: 78.7, 100) to 100.00 (95% CI: 100.0, 100.0) at primary level and 68.75 (95% CI: 50.0, 87.5) to 93.8 (95% CI: 86.7, 100.0) at secondary level. The median interval from symptom onset to first health contact fell by 9.3% (from 27.0 days (95% CI: 16.0, 33.3) to 24.5 days (95% CI: 15.0, 32.0)), and the time from first contact to confirmed diagnosis decreased by 54.2%. Treatment initiation interval increased by 11.9%, reflecting ongoing infrastructural constraints. Qualitative findings underscored the roles of supportive leadership, diagnostic supply limitations, cultural beliefs, and referral coordination in shaping outcomes. This quasi-experimental pre-post design without a control group limits strong causal inference, especially in the presence of potential confounders like parallel public-health initiatives and seasonal variations in care-seeking.

**Conclusions:**

A context-tailored, multilevel training and mentorship model was associated with improved provider capacity and reduced diagnostic delays in Northwest Ethiopia. While the initiative demonstrated high fidelity and adaptability in conflict-affected settings, achieving timely treatment requires further investment in diagnostic infrastructure. These tools and protocols are well-positioned for national scale-up and integration into routine continuing medical education.

## Author summary

**Why was this study done?:**

- Childhood cancer survival in low-income countries is low due to delayed diagnosis.
- In Northwest Ethiopia, limited provider training, weak referral systems, and low community awareness contribute to diagnostic delays.
- Interventions addressing early diagnosis across all health-system levels are scarce.

**What did the researchers do and find?:**

- A quasi-experimental pre–post mixed-methods study was conducted from January 2024 to October 2024 across three tiers of care.
- The intervention trained 18 secondary- and tertiary-level clinicians, 29 primary-level providers, and 1,020 health extension workers, followed by 6 months of mentorship.
- Median provider knowledge scores increased 54.6 to 90.9, and practice proficiency increased 87.5 to 100 at primary-level health providers.
- Median provider knowledge scores increased 36.4 to 90.9, and practice proficiency increased 68.8 to 93.8 at secondary-level health providers
- The median interval from symptom onset to diagnosis decreased by 54.2%, and patient delay decreased by 10.3%.

**What do these findings mean?:**

- Multilevel onsite training and mentorship can strengthen early childhood cancer diagnosis in low-resource settings.
- Persistent treatment delays indicate the need for investment in diagnostic and treatment capacity.
- The absence of a control group limits causal interpretation.

## Abstract 3

In this quasi-experimental mixed method study, Mulugeta Ayalew Yimer and colleagues investigate if a multilevel onsite training and mentorship model could improve early recognition, referral practices, and timely diagnosis of childhood cancer in Northwest Ethiopia.

## Introduction

Childhood cancer is an increasing global public health concern, with an estimated 300,000 new cases diagnosed annually, most of which occur in low- and middle-income countries [1]. Survival in low-income countries is dismal (<30%) compared to >80% in high-income countries [2,3]. Ethiopia faces this challenge acutely: a recent registry study notes that most Ethiopian children present with advanced-stage cancer and poor prognosis [4,5]. In fact, no pediatric oncology center existed in Northwest Ethiopia until 2019, and local data show very late presentation (e.g., over one-third of Wilms tumor cases at Gondar Hospital were stage IV at diagnosis [6]).

Key contributing factors such as low public awareness, a severe shortage of oncology specialists, inefficient referral systems, and prolonged diagnostic delays (often months) underscore the urgent need for intervention [2,3]. These baseline realities of high child cancer burden, very low survival, and systemic delays in Northwest Ethiopia define the critical problem that this project addresses.

The literature confirms that low-resource settings suffer disproportionately from childhood cancer mortality due to late diagnosis and limited services. Globally, ≈90% of childhood cancers occur in low- and middle-income countries, yet survival in these countries remains below 30% [7,8]. Many studies in sub-Saharan Africa report long diagnostic delays, a high percentage of advanced-stage presentation, and multiple barriers at the family, provider, and system levels. For example, the Addis Ababa registry found that nearly all Ethiopian children were diagnosed at late stages [9,10]. Previous efforts to improve early diagnosis include World Health Organization (WHO)/International Society of Paediatric Oncology (SIOP) campaigns (e.g., the CureAll framework) and educational workshops (e.g., a 2016 SIOP workshop in Jimma), which highlighted training and awareness as priorities [11].

Late diagnosis of childhood cancer remains a critical obstacle to improving survival in low-income settings [12,13]. In Northwest Ethiopia, children often arrive at advanced stages due to frontline providers’ limited training in early detection and families’ delayed care-seeking from low awareness or cultural beliefs. To address this, our University of Gondar team launched a capacity-building initiative in September 2023. This study aimed to enhance provider skills across all healthcare levels, improve referrals, and involve health extension workers (HEWs) in outreach driven by preventable treatment failures and a commitment to equitable pediatric oncology care.

In Ethiopia, partnerships like The Aslan Project have established pediatric oncology units, but few programs focus on early detection. Clinician and community training has improved cancer recognition through workshops in Tanzania and Uganda cut diagnostic delays by 40%, while Kenya’s campaigns boosted caregiver awareness [14–16].

Implementation research using the Consolidated Framework for Implementation Research (CFIR) highlights the importance of leadership engagement, adaptable training materials, and data feedback loops for sustaining practice change. However, few initiatives have integrated all three tiers of care facility, referral network, and community outreach in a coherent, onsite model tailored to local workflows. The existing studies underscore the heavy burden and complex delays in sub-Saharan child cancer care, and they point to training and health-system strengthening as key interventions [17–19].

Our intervention drew on CFIR to shape both design and evaluation. This intervention was designed on the premise that focused; multi-tiered training can overcome known barriers to early diagnosis. The WHO’s “CureAll” initiative and related frameworks emphasize strengthening health worker skills and referral networks as critical enablers of early childhood cancer care [2]. We also drew on the well-established “Three-Delay” model (adapted for pediatric oncology) which posits that delays arise at (1) the decision to seek care, (2) reaching a facility, and (3) receiving timely diagnosis once at a facility. By educating providers at multiple levels (from peripheral health centers to referral hospitals) and engaging communities, the intervention addresses each of these levels: improving symptom awareness (delay 1), streamlining referral paths (delay 2), and enhancing diagnostic capacity (delay 3). In practice, the project provided hands-on onsite training, case discussions, and referral protocols—methods grounded in adult learning theory and prior successes (e.g., training sessions conducted by The Aslan Project in 2012–2016) [20].

It was assumed that hands-on, context-specific training at each level would be more effective than offsite lectures and that embedding mentorship within existing supervisory structures would reinforce skills. The pictorial manuals for HEWs were based on adult‐learning theory, using visuals to overcome literacy barriers and to facilitate peer‐to‐peer teaching. Strengthening inter‐facility communication through standard referral forms and monthly case conferences was intended to reduce system fragmentation. Underpinning these choices was the belief that combining technical capacity‐building with supportive leadership and community engagement would accelerate diagnostic pathways and ultimately improve survival. In sum, the intervention was expected to work in this context because it directly targets the documented knowledge and system gaps identified in Ethiopia, leveraging international collaborations and WHO-endorsed strategies to accelerate early cancer detection.

The purpose of this project was to improve early diagnosis of childhood cancer in Northwest Ethiopia by building the capacity of healthcare providers through a multilevel onsite training intervention. Specifically, the project aimed to strengthen health workers’ knowledge and skills to recognize cancer warning signs, streamline referral pathways, and diagnose pediatric cancers sooner than current practice. This report presents the rationale, methods, and results of that intervention. It details how the training was implemented, and evaluates whether it achieved the goal of increasing timely diagnosis (for example, by raising provider awareness and referrals). In doing so, the report provides a clear account of the project’s objectives and outcomes, serving both to document the intervention’s impact and to inform future quality improvement efforts in similar low-resource settings.

## Methods

### Study setting

The University of Gondar Comprehensive Specialized Hospital (UoG-CSH) is the only pediatric haemato-oncology center serving a catchment of some 60 million people—20 million of whom are children under 19 years within a 350 km radius of Gondar City, Ethiopia. Its pediatric haemato-oncology unit comprises 35 inpatient beds, a procedure room, and dedicated medication-preparation facilities. The specialist team includes one pediatric haemato-oncologist, three haemato-oncology fellows, pediatric residents, nurses, a nutritionist, and a clinical psychologist. Peripheral facilities consist of 14 primary and general hospitals, 68 primary health centers, and 340 health posts staffed by health officers, general practitioners, nurses, and 1,020 HEWs. Before this project, more than 75% of frontline clinicians reported no formal training in childhood cancer, and baseline audits found a median total delay of 69 days from symptom onset to treatment initiation 27 days of patient delay and 56.5 days of diagnostic delay.

The study was conducted in the Amhara Region of northern Ethiopia, an area heavily impacted by active, non-international armed conflict since April 2023. Hostilities between the Ethiopian National Defense Force (ENDF) and the regional Fano militia have caused widespread instability, telecommunication shutdowns, and severe movement restrictions across local administrative zones. This volatile, conflict-affected environment created distinct operational constraints that directly shaped data collection protocols and participant accessibility.

**Interventions:** The intervention unfolded in three tiers, all delivered onsite between January 2024 and September 2024.

**Level I (secondary- and tertiary-level healthcare):** General practitioners, pediatricians, and nurses participated in a 10-day immersive training covering epidemiology, clinical presentation of common hematologic and solid tumors, diagnostic test interpretation, and case-based discussions. Training materials included slide decks, local-language pictorial manuals, and clinical attachments in the UoG-CSH unit.

**Level II (primary-level healthcare):** Health officers and nurses underwent a seven-day course focusing on warning signs, risk stratification, referral protocols, and use of standardized referral forms. The curriculum emphasized practical exercises and local adaptation of materials to primary-care workflows

**Level III (Health Extension Workers):** 1,020 HEWs received five days of training using a pictorial photo-book translated into local terminology (e.g., “nififit” for lymphadenopathy). Role-plays and community-engagement planning equipped HEWs to conduct home-to-home awareness and referral mapping.

All tiers then engaged in a 6-month mentorship phase combining monthly on-site supervision by a multidisciplinary team (haemato-oncologist, pathologist, clinical oncologist, public health specialists) with tele-support, case reviews, and rapid feedback loops to reinforce learning and streamline inter-facility communication. The mentors were general practitioners, nurses, health officers, and HEWs who received training from teams composed of pediatric haemato-oncologists (PHOs), haemato-oncology fellows, pediatric residents, nurses, a nutritionist, and a clinical psychologist. Those responsible for remote supervision were general practitioners, nurses, and HEWs who had been trained by the mentors (S1 File).

### Study of the intervention

A quasi-experimental, pre-post mixed-methods design assessed intervention impact. Quantitatively, Knowledge-Attitude-Practice (KAP) surveys were administered immediately before training and again 6 months afterward. Patient-journey intervals (symptom onset to first contact; first contact to confirmed diagnosis; diagnosis to treatment) were extracted from 100 pediatric oncology charts at baseline and 6 months post-implementation, which are all available charts during the study period. Qualitative data were collected through semi-structured interviews with 18 Level I and 29 Level II participants and focus groups with HEWs, probing barriers and facilitators using CFIR).

To attribute outcomes to the intervention rather than secular trends, post-intervention measures were compared to the same facilities ‘historical data over the prior year and triangulated quantitative findings with qualitative insights, seeking convergence across data sources.

The qualitative component explored healthcare providers’ and stakeholders’ experiences with the multilevel onsite training and mentorship model for early childhood cancer diagnosis in Northwest Ethiopia. Purposive sampling was used to recruit healthcare providers, mentors/trainers, and facility managers with ≥6 months of experience from intervention facilities, excluding purely administrative staff, and continued until data saturation was achieved. Data were collected through semi-structured, pre-tested in-depth interviews (IDIs) and focus group discussions (FGDs) conducted in private settings, lasting 45–70 min, audio-recorded with consent, supplemented by field notes, and transcribed and translated into English. Thematic analysis followed an inductive approach, with independent coding by multiple researchers, resolution of discrepancies through discussion, and iterative refinement with reflexive memos. Potential biases included social desirability, researcher familiarity, and selection bias, mitigated through neutral probing, triangulation, and consensus coding. This study is reported as per the Consolidated criteria for reporting qualitative research (COREQ): a 32-item checklist for interviews and focus groups (S2 File).

### Measures

**Fig 1:**
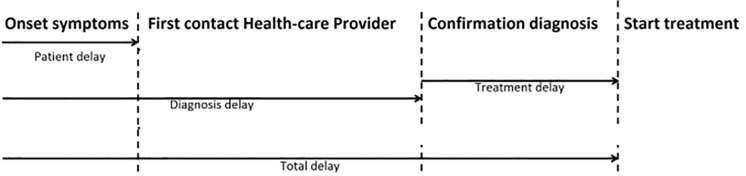
Types of delay (Patient delay = first contact healthcare provider minus onset symptoms, Diagnosis delay = confirmation of diagnosis minus onset of symptoms, Treatment delay = start treatment minus confirmation diagnosis, and Total delay = Start treatment minus onset symptoms).

KAP surveys comprised 15 multiple-choice knowledge items, eight attitude Likert-scale statements, and four practice Likert-scale items. Scores were normalized to a 0–100 scale using standard formulas. Primary outcomes were median changes in KAP scores. Secondary outcomes aligned with WHO benchmarks: proportion of patients with symptom-to-first contact under 30 days, diagnosis confirmation within 30 days of first contact, and treatment initiation within 30 days of diagnosis. Patient-journey intervals definitions: days from symptom onset to histological confirmation and days from referral decision to tertiary consultation. Data completeness was monitored via double-entry checks, 10% monthly chart audits, and predefined rules for handling missing values (listwise deletion if <5% missing; multiple imputation otherwise). Details of the types of delays are explained in the figure below (Fig 1 and S3 File).

### Operational definition

**Three tiers of care:** In Ethiopia, the three tiers of care mean a structured health system with Primary (Health Posts, Health Centers, Primary Hospitals for basic/preventive care), Secondary (General Hospitals for broader services), and Tertiary (Specialized Hospitals for complex issues) levels, linked by a referral system for efficient, accessible care, with primary healthcare as the main entry point.

**Knowledge:** A total of 15 multiple-choice questions, each with four options, were used to assess the participants’ knowledge. The equation used to calculate the normalized score is as follows: $\text{Normalized Knowledge Score}=(\frac{\text{Actual Score}-\text{4}}{\text{15}-\text{4}})$ *100

**Attitude:** A total of 5 Likert scale questions were used to assess the attitudes of study participants. All scores will be normalized to a 0–100 numeric rating scale using the following equation to calculate the normalized score. $\text{Normalized Attitude Score}=(\frac{\text{Actual Score}-\text{5}}{\text{40}-\text{5}})$ *100

**Practice:** A total of 4 Likert scale questions were used to assess the practices of study participants. The equation used to calculate the normalized score is as follows. $\text{Normalized practice Score}=(\frac{\text{Actual Score}-\text{4}}{\text{20}-\text{4}})$ *100

### Ethics approval and consent to participate

The University of Gondar Institutional Review Board approved the study (Protocol SOM/534/2023). All participants provided written informed consent. Patient data were de-identified per ethical guidelines and HEWs and clinical staff were assured that participation (or non-participation) would not affect employment. No conflicts of interest were declared by the implementation team.

### Analysis

Stata 17 was used for quantitative data analysis. Descriptive statistics (medians, interquartile ranges) characterized delays and KAP distributions. The Wilcoxon signed-rank test compared pre- and post-training KAP scores and interval changes. Kruskal–Wallis tests evaluated differences across facility levels and cancer types.

Qualitative transcripts were coded in NVivo using an a priori CFIR codebook augmented with inductive themes. Two analysts (AAT and ZA) independently coded transcripts, reconciled differences, and synthesized findings to contextualize quantitative results and explain variation in intervention uptake.

## Results

The study enrolled 18 Level I clinicians (general practitioners, pediatricians, nurses) and 29 Level II providers (health officers, nurses), along with one hundred pediatric patients with cancer records. The study achieved a 100% response rate from all participants.

### Level 1 Participants (secondary and tertiary level healthcare)

The mean age of the study participants was 30.88 ± 3.04 (SD). None of the study participants received any type of training on childhood cancer after graduation or deployment. More than three-fourths of the study participants were from primary hospitals (Table 1).

**Table 1:** Description of sociodemographic characteristics of study participants level 1.

Variables		Frequency (*N* = 18)	Percentage (%)
Age (*N* = 17)		30.88 ± 3.04 (Std)	
Sex	Male	14	77.78
	Female	4	22.22
Education level	BSc	1	5.56
	MSc/MPH	1	5.56
	Medical doctorate	14	77.78
	Other*	2	11.10
Experience in years		2.16 ± 1.65 (Std)	
Health institution	Health center	2	11.10
	Primary hospital	14	77.78
	General hospital	1	5.56
	Tertiary hospital	1	5.56
Prior training on childhood cancer	No	18	100%

^N^.B.

BSc, Bachelor of Sciences; MSc, Master of Sciences; MPH, Master of Public Health.

*Certificate and BA (Bachelor of Arts).

### Level II participants (primary level healthcare)

The mean age of the study participants was 29.42 ± 3.28 (Std). Most of the study participants did not receive any type of training on childhood cancer after graduation or deployment. Most of the study participants were from health centers (Table 2).

**Table 2:** Description of sociodemographic characteristics of study participants’ level II.

Variables		Frequency (*N* = 29)	Percentage (%)
Age (*N* = 26)		29.42 ± 3.28 (Std)	
Sex	Male	16	55.20
	Female	13	44.80
Education level	Certificate	1	3.40
	BSc	28	96.60
Experience in years		4.80 ± 2.30 (Std)	
Health institution	Health center	28	96.60
	Primary hospital	1	3.40
Prior training on childhood cancer	No	25	86.20
	Yes	4	13.80

^N^.B.

**Certificate:** Certificate refers to individuals who have completed Grade 10 and undertaken 1-year training programs.

**BSc:** Bachelor of Sciences.

#### Multilevel onsite training intervention.

The intervention commenced in January 2024 with a 10-day Level I training for 18 hospital-based clinicians, followed in February by a seven-day Level II workshop for 29 primary-care providers, and in March by a five-day pictorial training module for 1,020 HEWs. From April through September 2024, a 6-month mentorship phase combined monthly onsite visits with tele-support. Although these core activities adhered to the original timeline, two adaptations were introduced in June 2024. First, civil unrest in certain districts and associated travel restrictions had prevented many HEWs from attending the sessions in March, 2024. To address this gap, cascade ‘mini-trainings’ were organized at nearby secondary hospitals, each serving five adjacent health posts, led by Level I and II graduates under the research team’s supervision. These sessions restored HEW participation to over 80% in previously unreachable areas. Second, heavy seasonal rains in July disrupted travel and delayed several mentorship visits; in response, the frequency of tele-support calls was increased from monthly to biweekly, ensuring uninterrupted supervisory contact.

### Process measures and outcomes

**Fig 2:**
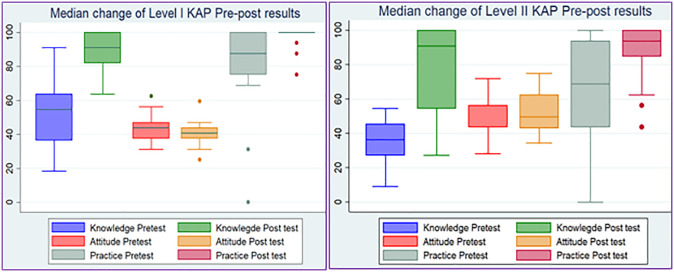
A box plot illustrating the difference between pre- and post-intervention measurements (In the box plots, the center line represents the median; the lower and upper box limits indicate the first (Q1) and third (Q3) quartiles; and the whiskers extend to the minimum and maximum values. The y-axis refers to the knowledge, attitude, and practice scores out of 100.

Process monitoring showed high fidelity: 90% of planned training days were delivered, and 87% of participants attended all sessions. Monthly mentorship logs documented an average of 25 case‐review discussions per month and rapid feedback loops on referral form completion. Outcome measures demonstrated substantial gains. Median knowledge scores at Levels I and II rose by 54.6 (95% CI: 36.4, 63.6) to 90.9 (95% CI: 81.8, 100.0) and 36.4 (95% CI: 27.3–45.5) to 90.9 (95% CI: 61.4, 93.7) points, respectively. Median practice proficiency increased from 87.5 (95% CI: 78.7, 100.0) to 100 (95% CI: 100.0,100.0) at Level I and from 68.8 (95% CI: 50.0, 87.5) to 93.8 (95% CI: 86.7, 100.0) at Level II. Unfavorable attitude scores declined modestly at both levels (Level I: 43.8 (95% CI: 37.5–46.9) to 40.6 (95% CI: 37.5, 42.8) and Level II: 56.3 (95% CI: 48.1, 56.3) to 49.6 (95% CI: 46.3, 56.2)) (Fig 2 and S4 File).

Among HEWs, referrals of suspected cases tripled (from 0.4 to 1.2 referrals per HEW per month) as recorded in district health‐information registers. In terms of patient journey outcomes, chart reviews of 100 patients with cancer revealed a baseline median total delay of 69.0 (IQR = 91.5) days, comprising a 27.0 (IQR = 49.00) days patient delay, 56.5 (IQR = 87.50) days diagnostic delay (Table 3). Six months post-intervention, patient delay decreased by 9.3% (to 24.5 days, *p* = 0.02), the diagnostic interval decreased by 54.3% (to 25.8 days, *p* < 0.001), and the interval from diagnosis to treatment initiation increased by 11.9% (to 15.9 days, *p* = 0.15). Treatment initiation at the oncology center accelerated from a median of 69.0 to 15.9 days (−76.9%) (Tables 3 and S1).

**Table 3:** Main indicators change of intervention.

Interval Description	Pre-Intervention Median (days) (IQR)	Post-Intervention Median (days) (IQR)	% Change	*Z*	*P*-value
Symptom onset → first medical contact (Patient Delay)	27 (IQR = 49)	24.5 (IQR = 44.5)	−9.3%	0.53	0.02
Symptom onset → diagnosis at first medical contact (Diagnostic Delay)	56.5 (IQR = 87.5)	25.8 (IQR = 40.3)	−54.2%	1.41	0.001
Diagnosis at oncology center → treatment initiation (Treatment Delay)^a^	69 (IQR = 91.5)	15.9 (IQR = 21.1)	−76.9%^a^	−0.55	0.15

^a^Treatment delay here reflects the interval from diagnosis at the oncology center to treatment start; the reduction is driven by faster post-diagnosis processes even as that interval remained the longest single segment of the care pathway.

### Contextual interactions

Several contextual factors shaped implementation. Civil unrest and security checkpoints in parts of the catchment area hindered patient referrals and HEW attendance at initial workshops. Seasonal rains in June–July 2024 disrupted travel, prompting increased tele-support. Concurrent public-health campaigns (e.g., malaria control) temporarily reallocated HEW efforts until cascade trainings resumed their focus. CFIR-guided qualitative analysis identified multiple facilitators and barriers. In our catchment area, several facility-level factors either enabled or impeded early diagnosis and treatment. Supportive hospital leadership and the presence of Electronic Community Health Information (ECHIS), District Health Information System (DHIS-2), and Electronic Medical Record (EMR) accelerated adoption of referral protocols and rapid feedback loops, whereas recurrent shortages of reagents and frequent staff rotations at peripheral sites undermined consistent practice of new skills. At the community level, the trusted status of health extension workers and the strong influence of local kebele structures (including religious and women’s associations) facilitated caregiver education and referral uptake. However, persistent beliefs that childhood cancer is caused by curses, widespread reliance on traditional healers, and low baseline awareness among caregivers continued to delay initial presentation. System-wide, the existing tiered referral hierarchy and growing Ministry of Health support provided a foundation for streamlining patient pathways, even as fragmented referral chains, limited insurance coverage, and intermittent transport availability posed important obstacles to achieving timely diagnosis and treatment.

### Associations between Interventions and outcomes

A total of 422 health facilities received mentorship (14 primary and general hospitals, 68 health centers, and 340 health posts), biweekly tele-support plus extra onsite visits, retained higher knowledge at 6 months (median 95.2% versus 88.3%, *p* = 0.04) and reduced referral-form errors by 60% versus 35% at standard-support sites (*p* < 0.01). HEWs trained via cascade sessions averaged 1.5 referrals per month, compared to 0.8 in areas without these catch-up trainings (*p* = 0.02), demonstrating the power of peer-led adaptation in a conflict zone.

### Unintended consequences

An unanticipated benefit was strengthened interdisciplinary collaboration: Of 1,067 participants, 747 (70%) reported that joint oncology case conferences fostered teamwork extending to other child-health areas, such as pediatric malaria. The modest decline in attitude scores suggests that future training must integrate motivational and cultural components alongside technical content. Twenty (1.96%) HEWs reported that the additional workload of cancer education detracted from time spent on other health priorities, prompting us to advocate for dedicated “cancer‐education days” to balance responsibilities.

### Missing data

Data completeness was high overall: KAP survey response rates were 98% pre-intervention and 95% post-intervention. Five patient charts (5%) lacked precise symptom onset dates and were excluded from patient interval analyses. For diagnostic intervals, 3% of records were missing histopathology confirmation dates; these cases were analyzed using multiple imputation, which produced similar median estimates, supporting the robustness of our findings. No referral form data were missing due to the mentorship team’s monthly audits and immediate corrections.

## Discussion

The multilevel onsite training and mentorship initiative markedly strengthened early childhood cancer diagnosis in Northwest Ethiopia. Clinicians at secondary and tertiary hospitals increased their median knowledge scores from 54.6 to 90.9 and from 36.4 to 90.9, respectively, while median practice proficiency rose to 93.8 and 100 at level I and level II, respectively. Health extension workers tripled their monthly referrals of suspected cases, and the median diagnostic interval declined by 54.2%, from 56.5 to 25.8 days, with a 10.3% reduction in patient delay (27.0 to 24.5 days). These findings align directly with our rationale grounded in adult learning principles and the three delay model and our specific aims to build provider capacity and expedite diagnosis [21,22].

A notable strength of the project was its seamless integration across the health system’s three tiers. By combining immersive workshops for hospital‐based clinicians, pictorial and role-play modules for over a thousand community health workers, and sustained 6-month mentorship including adaptive “mini‐trainings” in conflict-affected zones, this comprehensive strategy successfully enabled the intervention to maintain high fidelity (attendance > 95%) and achieve a broad reach. Embedding training and mentorship within existing supervisory and referral structures maximized relevance, reinforced learning, and fostered institutional ownership.

The temporal concordance between intervention delivery and improved outcomes, together with dose–response patterns where facilities receiving biweekly tele support and extra onsite visits retained higher knowledge (95.2% versus 88.3%) and sharply reduced referral-form errors (60% versus 35%), supports a potential causal relationship between our activities and the observed gains. Compared with similar efforts in Tanzania and Uganda, which achieved 30%–40% reductions in diagnostic delays through clinician workshops alone, our 54% decrease likely reflects the added impact of engaging community health workers and formalizing referral protocols [16,23–25].

Beyond quantitative improvements, the intervention catalyzed broader system-level effects. Seventy percent of participants reported that oncology case conferences fostered interdisciplinary collaboration, spilling over into pediatric malaria management and other child‐health domains. These synergies underscore how focused quality improvement efforts can strengthen health‐system resilience.

Contextual realities civil unrest, seasonal rains, and competing health campaigns tempered some anticipated outcomes. Despite the rise in knowledge and referral rates, tertiary center continued to face capacity challenges, which were further strained by increased referral volumes. The slight decline in attitude scores also indicates that technical training alone was insufficient to shift deeper cultural and motivational barriers.

From a resource standpoint, the entire intervention was executed within the USD 52,762 budget, demonstrating that strategic investment in capacity building can yield substantial returns without prohibitive costs. The primary opportunity cost was diverting HEW time from other campaigns, this was partially mitigated by scheduling dedicated “cancer education days” during cascade trainings.

As a limitation, our quasi-experimental, pre-post design without a contemporaneous control group limits strong causal inference, particularly given potential confounders such as parallel public‐health initiatives and seasonal variations in care‐seeking. Although contextual factors were tracked and sensitivity analyses conducted, residual bias from unmeasured influences cannot be excluded. Measurement imprecision arising from chart reviews was addressed through double data entry, monthly audits, and multiple imputation for the < 3% of missing time interval data; nonetheless, some misclassification may persist.

Generalizability may be constrained to settings with similar tiered referral architectures and community health worker networks. Regions lacking formal supervisory linkages or facing more severe security challenges may need tailored approaches. Finally, our attitude measures based on Likert scales may not fully capture complex motivational dynamics, suggesting a need for richer qualitative exploration in future studies. In addition, limited transferability, reliance on self-reported experiences, and possible loss of nuance during translation.

As a conclusion, this study demonstrates that a contextualized, multilevel onsite training and mentorship model can substantially enhance early childhood cancer diagnosis in low-resource settings. The intervention’s success is driven by concurrent gains in provider capacity, shortened diagnostic timelines, and strengthened interdisciplinary collaboration, which collectively demonstrate the model’s operational viability. Sustainability is supported by the Ministry of Health’s commitment to integrate the curricula into national Continuing Medical Education and by university led tele-support.

The model’s potential for spread is high in contexts with community health-worker platforms and tiered referral systems; adaptation may require integrating culturally tailored motivational modules and bolstering diagnostic infrastructure to reduce treatment initiation delays. Implications for practice include embedding regular mentorship, using cascade trainings for remote or conflict-affected areas, and formalizing referral pathways. Further research should assess long-term survival impacts, explore cost effectiveness in diverse environments, and develop strategies to sustain provider motivation.

## Supporting information

S1 File Research implementation protocol. (PDF)

S2 File COREQ checklist. (PDF)

S3 File Assessment Tool. (DOCX)

S4 File KAP pre- and post-test data. (XLS)

S1 Table Mean difference between stages of cancer and types of interventions. (DOCX)

## Abbreviations

CFIR: Consolidated Framework for Implementation Research
COREQ: Consolidated criteria for reporting qualitative research
ENDF: Ethiopian National Defense Force
FGDs: focus group discussions
HEWs: health extension workers
IDIs: in-depth interviews
KAP: Knowledge-Attitude-Practice
PHOs: pediatric haemato-oncologists
SIOP: International Society of Paediatric Oncology
UoG-CSH: University of Gondar Comprehensive Specialized Hospital
WHO: World Health Organization
