# Supplementary material for: Multilevel onsite training and mentorship model to accelerate early childhood cancer diagnosis in Northwest Ethiopia: A quasi-experimental mixed method study
Source: PLoS Med. 2026 Jun 29;23(6):e1005132. doi: 10.1371/journal.pmed.1005132 (PMC13313369; doi:10.1371/journal.pmed.1005132)
Supplement: S1 File — (PDF) [file pmed.1005132.s001.pdf]

Background and objective of the project:

Childhood cancer is emerging as a significant public health challenge globally, with a substantial burden in low and middle-income countries (LMICs). According to the World Health Organization (WHO), 400,000 new cases of cancer in children under 19 years are reported annually, resulting in 80,000 cancer-related deaths worldwide. Alarmingly, around 90% of these cases occur in LMICs. Ethiopia, in particular, faces the impact of childhood cancer, contributing to 15% of the national cancer burden.

The overall survival rates for childhood cancer in high-income countries (HICs) have reached 80%. However, in LMICs, including Ethiopia, less than 30% of cases are cured. One major contributor to this disparity is the delay in diagnoses, with advanced disease at presentation significantly impacting mortality rates. Ethiopia reports a considerably higher incidence of advanced disease compared to HICs and even other LMIC counterparts.

Childhood cancer accounts for a substantial portion of the national cancer burden in Ethiopia, with high rates of advanced disease at presentation. The delay in diagnosis is multifactorial, involving parental characteristics, healthcare system challenges, and clinical complexities. The median diagnosis latency is alarmingly high, emphasizing the critical need for interventions to expedite the diagnostic process. The delay in diagnosis, coupled with late referrals among pediatric cancer patients in Ethiopia, has led to prolonged diagnosis latency and, subsequently, increased childhood mortality rates.

In response to this critical issue, the research project, "Capacity Building Through Multilevel Onsite Training for Early Cancer Diagnosis in Ethiopia," aims to address these challenges by enhancing the capacity of healthcare professionals through targeted training and support.

Objectives:

Overall Goal: The overarching goal of the project is to improve early cancer diagnosis in Ethiopia, specifically focusing on childhood cancer. This aligns with the broader objective of strengthening oncology capacity in Sub-Saharan Africa.

The primary objectives are to:

1. Enhance the capacity of health professionals for early childhood cancer detection.
2. Increase the number of childhood cancer cases diagnosed at an early stage.
3. Address the specific challenges contributing to delayed diagnoses in the Ethiopian context.

Target Audience:

The project's primary focus is on building the capacity of community health workers, health officers, nurses, general practitioners, and pediatricians. Given the large catchment area and limited specialized pediatric oncology centers, the project aims to empower healthcare professionals at various levels to contribute to early cancer detection. The diverse training approach considers the unique roles and responsibilities of each target group, ensuring a tailored and effective intervention.

Project Design and Methods:

A pre-post mixed-method study will be employed to assess the impact of the training on healthcare professionals' capacity and the number of childhood cancer cases diagnosed at an early stage. The study design incorporates a baseline assessment of knowledge, attitudes, and practices, followed by targeted training and mentorship. The intervention is structured into three levels, each catering to the specific needs of the target audience. The training content covers epidemiology, clinical signs and symptoms, diagnostic tests, and early referral strategies.

Evaluation and Outcome:

The project's primary outcome is the improvement of health professionals' capacity, measured through changes in knowledge, attitudes, and practices related to childhood cancer diagnosis. Standardized questionnaires will be employed, with scores normalized for comprehensive evaluation. Secondary outcomes focus on the early diagnosis of childhood cancer, with key indicators including patient interval.

University of Gondar

Research Work Plan and Activities

Conceptualization Phase

1. **Letter of Intent (LOI) Development**
    - *Activities:*
    - **Initiation (July 01, 2022 - July 15, 2022):**
        - Form a preliminary team to oversee LOI development.
        - Outline key components of the LOI, including project goals and intentions.
            - Identify key components and structure.
            - Define the purpose and objectives of the LOI.
    - **Drafting (July 16, 2022 - July 25, 2022):**
        - Develop a preliminary version of the LOI document.
        - Elaborate on the project's significance and goals.
        - Clearly articulate the need for capacity building in early cancer diagnosis
    - **Stakeholder Engagement (July 26, 2022 - August 1, 2022):**
        - Present the draft to relevant stakeholders for input.
        - Gather feedback and suggestions for improvement.
    - **Refinement (August 2, 2022 - August 7, 2022):**
        - Revise the LOI based on stakeholder feedback.
        - Ensure clarity, conciseness, and alignment with project objectives.
        - Finalize the document for submission
2. **Proposal Development**
    - *Activities:*
    - **Framework Establishment (November 20, 2022 - November 25, 2022):**
        - Define the structure and components of the research proposal including background, objectives, methodology, and evaluation.
        - Establish research questions and hypotheses.
    - **Literature Review (November 26, 2022 - December 5, 2022):**
        - Conduct an extensive literature review on pediatric cancer in Ethiopia.
        - Investigate current challenges, gaps, and existing interventions.
        - Identify relevant studies, policies, and best practices.
    - **Develop Research Questions and Hypotheses:**
        - Formulate clear, research-focused questions.
        - Establish hypotheses to guide data collection and analysis.
    - **Methodology Design (December 6, 2022 - December 15, 2022):**
        - Design the research methodology, including training formats and evaluation methods.
        - Detail the approach to data collection and analysis.
    - **Drafting and Refinement (December 16, 2022 - December 30, 2022):**
        - Write each section with attention to detail.
        - Review and refine the proposal based on feedback.

Initiation Phase

3. **Regulatory Body and Contractual Agreement**
    - *Activities:*
    - **Contractual Agreements (March 8, 2023 - March 15, 2023):**
        - Draft contractual agreements with collaborating institutions.
        - Finalize and sign contracts, clarifying roles and responsibilities.
    - **Support Letter (March 01, 2023 - March 7, 2023):**
        - Identify relevant regulatory bodies for approval.
        - Initiate communication with regulatory bodies to get permission and support letter.
    - **Finalize and Sign Contracts:**
        - Facilitate signing between involved parties.
4. **Purchasing of Materials and Consumables**
    - *Activities:*
    - **Identify Required Training Materials and Consumables (March 22, 2023 - March 25, 2023):**
        - Compile a detailed list of necessities.

ጎንደር ዩኒቨርሲቲ University of Gondar
የሕክምና ትምህርት ቤት School of Medicine
College of Medicine & Health Sciences

        - Considerations for quality, quantity, and cost-effectiveness.
    - **Research Suppliers and Obtain Quotations (March 26, 2023 - April 3, 2023)::**
        - Reach out to potential suppliers.
        - Collect quotations for materials and consumables.
    - **Procure Materials and Consumables Within Budget Constraints:**
        - Evaluate quotations against the budget.
        - Make informed decisions on procurement.

5. **Document Preparations**
    - *Activities:*
    - **Questionnaire Development (March 21, 2023 - March 25, 2023):**
        - Develop a comprehensive questionnaire for health professionals.
        - Align questions with project goals and evaluation criteria.
    - **Training Materials (March 26, 2023 - April 5, 2023):**
        - Create a PowerPoint presentation for training sessions.
            - Structure content for effective knowledge transfer.
            - Include visuals, case studies, and interactive elements.
        - Compile a visually engaging photobook showcasing common childhood cancer symptoms.
            - Capture visual representations of symptoms.
            - Ensure cultural sensitivity and inclusivity.
6. **Questionnaire and Document Evaluation by Experts**
    - *Activities:*
    - **Expert Panel Identification (April 5, 2023 - April 10, 2023):**
        - Identify a panel of experts in pediatric oncology and training methodologies.
    - **Document Evaluation (April 11, 2023 - April 20, 2023):**
        - Distribute documents for expert evaluation.
        - Conduct a thorough review meeting to discuss feedback.
        - Incorporate expert recommendations into training materials.

Implementation Phases - Phase I

7. **Level I Training and Pre-Post Training Data Collection**
    - *Activities:*
    - **Training Planning (May 01, 2023 - May 5, 2023):**
        - Plan and organize Level I training sessions with a detailed curriculum.
        - Define the schedule and format.
        - Arrange logistics and venues.
    - **Pre-Training Assessment (May 6, 2023 - May 10, 2023):**
        - Conduct pre-training assessments to establish a baseline of participants' KAP.
        - Collect data on participants' current knowledge.
        - Tailor training content based on initial assessments.
    - **Training Implementation (May 11, 2023 - May 18, 2023):**
        - Engage expert trainers for effective sessions.
        - Implement Level I training, integrating detailed lectures and clinical attachments.
        - Provide hands-on experiences through clinical attachments.
    - **Post-Training Data Collection (May 19, 2023 - May 26, 2023):**
        - Collect post-training data to assess KAP enhancement and assess the immediate impact of the training.
        - Gather feedback from participants.
8. **Level II Training and Pre-Post Training Data Collection**
    - *Activities:*
    - **Training Planning (May 01, 2023 - May 5, 2023):**
        - Plan and organize Level II training sessions, focusing on health officers and nurses.
        - Ensure training content aligns with the specific needs of this group.
    - **Pre-Training Assessment (May 6, 2023 - May 10, 2023):**
        - Assess baseline KAP specific to primary care.
        - Adapt training content based on assessments
    - **Training Implementation (May 11, 2023 - May 18, 2023):**
        - Implement Level II training, emphasizing basic signs, symptoms, and early referral strategies.

University of Gondar
School of Medicine

        - Provide practical guidance for primary care scenarios.
    - **Post-Training Data Collection (May 19, 2023 - May 26, 2023):**
        - Collect post-training data to evaluate the impact of the training on participants.
        - Measure changes in knowledge and confidence.
        - Capture insights on the applicability of training in real-world scenarios.

9. **Level III Training and Pre-Post Training Data Collection**
    - *Activities:*
    - **Training Planning (May 01, 2023 - May 5, 2023):**
        - Plan and organize Level III training sessions for health extension workers.
        - Customize training content for community-level workers.
        - Consider cultural and linguistic nuances.
    - **Pre-Training Assessment (May 6, 2023 - May 10, 2023):**
        - Assess baseline knowledge and communication skills.
        - Tailor training materials to local contexts.
    - **Training Implementation (May 11, 2023 - May 25, 2023):**
        - Implement Level III training, emphasizing household-level awareness and early detection.
        - Foster engagement and facilitate discussions on community-specific challenges.
        - Focus on door-to-door outreach and early symptom recognition.
        - Develop training materials translated into local languages.
    - **Post-Training Data Collection (May 26, 2023 - June 29, 2023):**
        - Collect post-training data to measure the effectiveness of the training in this unique context.
        - Measure changes in awareness and practices.
        - Evaluate the effectiveness of localized training approaches.
10. **Mentorship/Supervision**
    - *Activities:*
    - **Schedule Establishment (May 10, 2023 - May 15, 2023):**
        - Establish **a** Mentorship Schedule for Continuous Support:
            - Define regular intervals for mentorship sessions.
            - Incorporate flexibility to address emerging needs.
        - Ensure availability and commitment from mentors.
    - **Regular Sessions (May 16, 2023 - December 30, 2023):**
        - Conduct regular mentorship/supervision sessions, addressing challenges and queries.
            - Create a supportive environment for open communication.
            - Address specific challenges faced by participants.
        - Guide participants in applying their newly acquired knowledge to real-world scenarios.
    - **Provide Guidance on Practical Application of Knowledge:**
        - Assist in translating training insights into daily practice.
        - Offer solutions for common issues encountered.
11. **Data Analysis and Presentation**
    - *Activities:*
    - **Data Cleaning (January 03, 2024 - January 05, 2024):**
        - Thoroughly clean and organize the collected data from pre-post assessments.
        - Address any discrepancies or missing information.
    - **Statistical Analysis (January 06, 2024 - January 12, 2024):**
        - Utilize statistical methods to analyze the data, highlighting trends and key insights.
    - **Presentation Development (January 13, 2024 - January 15, 2024):**
        - Develop visually appealing data visualizations for a comprehensive presentation.
        - Ensure clarity and accessibility for diverse audiences.
    - **Drafting (January 16, 2024 - January 17, 2024):**
        - Draft a detailed presentation summarizing the findings and lessons learned.
12. **Workshop 6-Month Project Evaluation**
    - *Activities:*
    - **Workshop Planning (January 27, 2024 - January 30, 2024):**
        - Plan and Organize a Workshop Involving All Stakeholders:
        - Facilitate collaboration and knowledge exchange.
        - Provide a platform for participants to share experiences.

University of Gondar
School of Medicine
College of Medicine & Health Sciences

    - Ensure participation from health professionals, mentors, and community representatives.
- **Facilitation (January 31, 2024 - February 1, 2024):**
    - Facilitate Discussions on Project Progress, Challenges, and Successes:
        - Encourage open dialogue on successes and setbacks.
        - Identify areas for improvement based on real-world experiences.
        - Encourage active participation and idea-sharing.
- **Feedback Collection (February 2, 2024 - February 5, 2024):**
    - Collect valuable feedback from participants to inform future improvements.
    - Document insights and recommendations for project enhancement.

Implementation Phases - Phase II

13. **Regular Mentorship/Supervision**

- *Activities:*
- **Continuation (February 01, 2024 - February 5, 2024):**
    - Continue the regular mentorship/supervision sessions established in Phase I.
    - Address emerging challenges and adapt training methodologies as needed.
- **Adaptations (February 6, 2024 - July 30, 2024):**
    - Foster a culture of continuous learning and application of knowledge.
    - Make any necessary adaptations based on ongoing evaluations.
- **Address Emerging Challenges and Adapt Training as Needed:**
    - Proactively identify challenges faced by participants.
    - Modify training content or approaches as necessary.
- **Encourage Continuous Learning and Application of Knowledge:**
    - Reinforce the importance of continuous improvement.
    - Promote a culture of learning and adaptation.

14. **Early Diagnosis Indicators Data Collection**

- *Activities:*
- **Data Collection Planning (February 01, 2024 - February 5, 2024):**
    - Plan and execute data collection activities to assess early diagnosis indicators.
    - Define early diagnosis indicators for assessment.
    - Develop protocols for consistent data collection.
- **Collect Early Diagnosis Indicators Data to Assess Practical Application:**
    - Measure changes in diagnostic intervals.
    - Assess disease stage at diagnosis post-training.
- **Data Analysis (February 6, 2024 - July 30, 2024):**
    - Analyze early diagnosis indicators to measure the impact of training.

Termination Phase

15. **Data Analysis, Write Up, and Dissemination**

- *Activities:*
- **Final Data Integration (August 01, 2024 - August 5, 2024):**
    - Finalize the data analysis, incorporating data from both Phase I and Phase II.
    - Ensure consistency and accuracy in data integration.
- **Research Paper Drafting (August 6, 2024 - August 10, 2024):**
    - Draft a comprehensive research paper highlighting key findings, lessons learned, and recommendations.
    - Collaborate with co-authors for diverse perspectives.
- **Dissemination Material Development (August 11, 2024 - August 15, 2024):**
    - Develop dissemination materials tailored for various stakeholders, including policymakers and healthcare professionals.
    - Ensure accessibility and relevance of materials.
- **Dissemination Activities (August 16, 2024 - September 02, 2024):**
    - Disseminate research findings through conferences, peer-reviewed publications, and targeted forums.
    - Submit articles to peer-reviewed journals.
    - Engage in targeted forums to reach specific stakeholders.

Sustainability and Extension Phase

16. **Supportive Supervisions and Tele Support Extension (August 16, 2024 - September 02, 2024):**

University of Gondar
School of Medicine
College of Medicine & Health Sciences

- *Activities:*
  - Extend supportive supervisions and tele support for an additional six months in identified areas to ensure sustained knowledge application.
  - Monitor the continued implementation of early diagnosis practices and address any challenges that may arise.

17. **Final Evaluation and Reporting (August 16, 2024 - September 02, 2024):**
- *Activities:*
  - Conduct a final evaluation to assess the number of cases diagnosed at an early stage in the extended areas.
  - Report and disseminate findings to concerned bodies, emphasizing the long-term impact and sustainability of the project.

University of Gondar
School of Medicine
College of Medicine & Health Sciences
