# Supplementary material for: Multilevel onsite training and mentorship model to accelerate early childhood cancer diagnosis in Northwest Ethiopia: A quasi-experimental mixed method study
Source: PLoS Med. 2026 Jun 29;23(6):e1005132. doi: 10.1371/journal.pmed.1005132 (PMC13313369; doi:10.1371/journal.pmed.1005132)
Supplement: S3 File — (DOCX) [file pmed.1005132.s003.docx]

**Level I and Level 2 training Pre-test**

| 1. **Training participants details** | | | | | |
| --- | --- | --- | --- | --- | --- |
| 1 | What is your age (in years)? | ______________ | | | |
| 2 | What is your gender? | 1. Male 2. Female | | | |
| 3 | What is the highest level of your education? | 1. Certificate 2. Diploma 3. Degree, BSc 4. MSC/MPH 5. MD 6. Other, specify _____________ | | | |
| 4 | What are the total years of your professional work? | ____________ | | | |
| **5** | Where do you work? | 1. Health Center 2. Primary Hospital 3. Tertiary Hospital | | | |
| **6** | Have you received any training on childhood Cancer after graduation/deployment? | 1. Yes 2. No | | | |
| 1. **Knowledge Questions** | | | | | |
| 1 | What is the most common childhood cancer worldwide and in Gondar? | 1. **Leukemia** 2. Retinoblastoma 3. Brain tumors 4. Lymphoma | | | |
| 2 | What is the most common childhood cancer in Gondar? | 1. **Leukemia** 2. Retinoblastoma 3. Brain tumors 4. Lymphoma | | | |
| 3 | What is the most common etiology of childhood malignancies? | 1. Genetic predisposition 2. Radiation exposure 3. Viral infection 4. **Unknown** | | | |
| 4 | Which of the following is **NOT** a predictor of mortality in childhood malignancy? | 1. Early diagnosis and treatment 2. Type of cancer 3. Quality of supportive care 4. **None** | | | |
| 5 | Which is the earliest manifestation of retinoblastoma in children? | **A. White-eye reflex**  B. Red-eye reflex  C. Proptosis  D. Eye discharge and squint | | | |
| 6 | A child with bimanually palpable, ballotable abdominal mass, hematuria, and hypertension should be suspected to have | 1. **Wilm's Tumor** 2. Hepatoblastoma 3. Lymphoma 4. Rhabdomyosarcoma | | | |
| 7 | What is the most common modality of treatment of childhood malignancies? | 1. Surgery 2. Radiotherapy 3. **Chemotherapy** 4. Stem cell therapy | | | |
| 8 | What is the most common site of neuroblastoma? | 1. **Abdomino-pelvic** 2. Thoracic 3. Cervical 4. Head and neck | | | |
| 9 | Which of the following is not early symptom of brain tumor? | 1. Subtle change in personality 2. Subtle change in mentation 3. Subtle change in speech 4. **Motor weakness** | | | |
| 10 | Which of the following is wrong CBC finding in children with acute leukemia? | 1. Hgb: Always low 2. **WBC: Always high** 3. Platelet: Low or normal 4. Blood smear: Blasts | | | |
| 11 | Which of the following characteristics of lymphadenopathy doesn’t indicate Hodgkin’s Lymphoma? | 1. Supraclavicular site 2. Size ≥ 2.5 cm 3. Duration ≥4 weeks 4. **Pain and tenderness** | | | |
| 12 | Which of the following is not a sign of oncologic emergency? | 1. Head and neck edema 2. Paraplegia 3. Neutropenic fever 4. **Hypouricemia and hypokalemia** | | | |
| 13 | Which of the following is the most common site of rhabdomyosarcoma | 1. **Head and neck** 2. Orbit 3. Extremities 4. Retroperitoneal | | | |
| 14 | What is the diagnostic test for Childhood cancer? | A. X-ray  B. Endoscopy  **C. Each type of Cancer has a different diagnostic test** | | | |
| 15 | Which of the following is true about the use of ultrasound in the diagnosis of caner? | 1. Identify origin of mass 2. Identify associated fluid   **C. Identify bone metastasis**  D. Direct needle aspiration | | | |
| 1. **Attitude Questions** | | | | | |
| 1 | Most childhood cancers occur in developing countries (LMIC). | 1. Strongly disagree 2. Disagree 3. Neutral | 1. Agree 2. Strongly agree | | |
| 2 | Most of childhood cancers are curable. | 1. Strongly disagree 2. Disagree 3. Neutral | 1. Agree 2. Strongly agree | | |
| 3 | Early diagnosis of childhood cancer has a huge survival benefit. | 1. Strongly disagree 2. Disagree 3. Neutral | | | 1. Agree 2. Strongly agree |
| 4 | The health of children with cancer is beyond the doctor's control and determined by luck or fate. | 1. Strongly disagree 2. Disagree | | | 1. Neutral 2. Agree 3. Strongly agree |
| 5 | Almost all children with cancer die. | 1. Strongly disagree 2. Disagree 3. Neutral | | | 1. Agree 2. Strongly agree |
| 6 | Alternative treatment/traditional medicine can cure cancer. | 1. Strongly disagree 2. Disagree 3. Neutral | | | 1. Agree 2. Strongly agree |
| 7 | The sun is rectangular in shape. | 1. Strongly disagree 2. Disagree 3. Neutral | | | 1. Agree 2. Strongly agree |
| 8 | Childhood cancer is preventable | 1. Strongly disagree 2. Disagree 3. Neutral | | | 1. Agree 2. Strongly agree |
| 1. **Practice Questions** | | | | | |
| 1 | In the past 6 month, have you diagnosed/suspected a childhood cancer. If no, skip to question number 4. | 1. Yes 2. No | | | |
| 2 | If yes, where did you refer the child to? | 1. Another Health Center 2. Primary hospital. 3. Tertiary hospital | | | |
| 3 | Did you receive feedback on your referral? | 1. Yes | | 1. No | |
| 4 | Once diagnosed, do you regularly follow patients with childhood cancer in your institute? | 1. Yes | | 1. No | |
| 5 | Is there a pediatric haemato-oncology center in your referral chains? | 1. Yes 2. No | | 1. I don’t know | |
| For the following questions, please indicate your level of comfort with each task from very uncomfortable (couldn’t do it on your own) to very comfortable (could teach someone else) | | | | | |
| 6 | Listing early manifestation of common childhood caner | 1. Very uncomfortable 2. Somewhat uncomfortable 3. Neutral 4. Somewhat comfortable 5. Very comfortable | | | |
| 7 | Conducting physical examination to identify early sings of common childhood cancer. | A. Very uncomfortable  B. Somewhat uncomfortable  C. Neutral  D. Somewhat comfortable  E. Very comfortable | | | |
| 8 | Order proper investigations for a child suspected with cancer. | A. Very uncomfortable  B. Somewhat uncomfortable  C. Neutral  D. Somewhat comfortable  E. Very comfortable | | | |
| 9 | Identifying patients with childhood cancer who need referral to Pediatric Haemato-Oncology center | A. Very uncomfortable  B. Somewhat uncomfortable  C. Neutral  D. Somewhat comfortable  E. Very comfortable | | | |
