# Supplementary material for: Multilevel onsite training and mentorship model to accelerate early childhood cancer diagnosis in Northwest Ethiopia: A quasi-experimental mixed method study
Source: PLoS Med. 2026 Jun 29;23(6):e1005132. doi: 10.1371/journal.pmed.1005132 (PMC13313369; doi:10.1371/journal.pmed.1005132)
Supplement: S1 Table — (DOCX) [file pmed.1005132.s005.docx]

Supplementary Table 1: Mean difference between stages of cancer and types of interventions.

|  | Kruskal-Wallis rank test  Stages of cancer | | | Wilcoxon ranked-sum test  Groups of intervention (pre & post) | |
| --- | --- | --- | --- | --- | --- |
|  | Chi-square | df | Sig. | Z | Sig. |
| Patient delay | 18.82 | 5 | <0.001 | 0.53 | 0.02 |
| Diagnostic Delay | 14.23 | 5 | 0.01 | 1.41 | 0.001 |
| Referral Delay | 15.10 | 5 | 0.01 | 1.24 | 0.22 |
| Tertiary Care Diagnostic Delay | 16.58 | 5 | 0.01 | -0.13 | 0.90 |
| Primary Care Delay | 18.04 | 5 | <0.001 | 2.64 | 0.01 |
| Physician Delay | 21.11 | 5 | <0.001 | -0.77 | 0.44 |
| Healthcare System Delay | 17.16 | 5 | <0.001 | -0.98 | 0.33 |
| Post-Diagnostic Referral Interval | 0.18 | 5 | 1.00 | 12.10 | <0.001 |
| Secondary Diagnostic Interval | 3.69 | 5 | 0.59 | 11.15 | <0.001 |
| Primary Care to Treatment Interval | 11.49 | 5 | 0.04 | 6.47 | <0.001 |
| Tertiary Care Diagnostic Delay | 5.03 | 5 | 0.41 | -8.74 | <0.001 |
| Referral to Treatment Interval | 6.51 | 5 | 0.26 | -6.69 | <0.001 |
| Treatment Delay | 22.01 | 5 | 0.00 | -0.55 | 0.15 |
| Total delay | 16.32 | 5 | 0.01 | -0.40 | 0.69 |
