# Supplementary material for: Multilevel onsite training and mentorship model to accelerate early childhood cancer diagnosis in Northwest Ethiopia: A quasi-experimental mixed method study
Source: PLoS Med. 2026 Jun 29;23(6):e1005132. doi: 10.1371/journal.pmed.1005132 (PMC13313369; doi:10.1371/journal.pmed.1005132)
Supplement: Attachment — Submitted filename: Authors response to editors_R3.docx [file pmed.1005132.s010.docx]

**Response Editors**

Dear Editors,

We would like to sincerely thank you very much for your time and invaluable comments and suggestions on our manuscript. We have carefully considered all your suggestions and comments, have revised the manuscript accordingly. Below, we have attached the authors' response to the editors’ comments and suggestions, indicated in ***Bold Italics***.

GENERAL
* We suggest changing the title to: “A Multilevel Onsite Training and Mentorship Model to Accelerate Early Childhood Cancer Diagnosis in Northwest Ethiopia: A Quasi-experimental mixed method study”

***Response to the Editors: Thank you very much for the suggestions. We have revised the title accordingly.***

* Please ensure that all abbreviations are defined at first use throughout the text. This also includes statistical abbreviations.

***Response to the Editors: Thank you for your comment. We have ensured that all abbreviations, including statistical abbreviations, are clearly defined at first use throughout the text.***

* Please ensure that tables and figures, including those in supplementary files, are appropriately referenced in the main text.

***Response to the Editors: Thank you for your comment. We have ensured that all tables and figures, including those in the supplementary files, are appropriately referenced in the main text.***

* Please define all abbreviations used in each figure or table in the corresponding figure description or below the table.

***Response to the Editors: We sincerely thank you for your valuable comments. All abbreviations have now been clearly defined.***

* Please remove descriptions such as “paediatric oncology patients” or “cancer patients” and use patient-centered language instead. Please note that patient-centered language is constructed with the use of post-modified nouns putting the person first in the sentence structure (e.g. “patients with cancer”).

***Response to Editors: Thank you very much for your valuable comment. We have revised the manuscript to use patient-centered language throughout. Descriptions such as ‘paediatric oncology patients’ or ‘cancer patients’ have been replaced with person-first terminology (e.g., ‘patients with cancer’).***

* The Data Availability Statement (DAS) requires revision. In the manuscript and the online submission form you have stated that “All relevant data is available within the manuscript and Supporting Information files”. However, in your last rebuttal you have stated that “the data are owned by a third party but are freely available upon request” and that you “have provided the owner of the dataset and the appropriate contact information for data requests”. Unfortunately, we cannot find an updated statement with the appropriate contact information. Please revise your Data Availability Statement to ensure it is accurate and complies with our requirements.

***Response to Editors: We sincerely thank you for your suggestions. The Data Availability Statement clearly indicated in the declaration section.***

* Thank you for providing your COREQ checklist. Please replace the text excerpts with paragraph numbers per section (e.g. "Methods, paragraph 1").

***Response to Editors: Thank you very much for the comments. We have corrected it accordingly.***

* Please note that you stated that you added a completed CROSS checklist as S2 file and added a statement in the Methods section mentioning the CROSS checklist. However, we can neither find the completed checklist nor the statement.

***Response to Editors: Thank you very much for the comments. We submitted the S2 file as the COREQ checklist, not the CROSS checklist. Therefore, we could not include the term CROSS checklist in the main document.***

* “Where data were available, we have added a comparison of key characteristics between survey respondents and nonrespondents in the Methods/Results section to assess potential nonresponse bias. Where such comparisons were not feasible, this has been clearly acknowledged as a limitation.” – We cannot seem to find any data on nonrespondents in the manuscript. Please comment.

***Response to Editors: Thank you very much for the comments. We include the respondent rate in the result section.***

* “The survey response rate has now been added to the Abstract to improve transparency and reporting completeness.” – It seems that this has not been included in the Abstract.

***Response to Editors: Thank you very much for the comments. We have included it.***

* “A complete copy of the survey instrument has been added to the Supplementary Files for reference and transparency.” – It seems this file has not been uploaded.

***Response to Editors: Thank you very much for the comments. Thank you for bringing this to our attention. The survey instrument has now been uploaded to the Supplementary Files (S3_File).***

ABSTRACT
Please note that the comments and questions we raised in our previous decision letter were added because these details are missing from the Abstract. Although we appreciate your detailed responses, it appears that you have not included the relevant information in the Abstract. Please carefully revise the Abstract, taking into account the comments below and from the previous decision letter.

* Abstract

Background: The final sentence should clearly state the study question.

***Response to Editors: Thank you very much for the comments. We have included the study questions.***

* Please note that a very brief definition of the three tiers of care needs to be in the Abstract.

***Response to Editors: We sincerely thank you for your valuable comments. The relevant details have now been incorporated into the Methods section under the ‘Operational Definitions’ subsection.***

* “A six-month mentorship program combined monthly onsite visits with remote supervision.” – This statement still lacks detail. Please describe who was in the mentor group and who the mentees were and what remote supervision entails.

***Response to the editors: Thank you very much for your comments. The mentors were general practitioners, nurses, health officers, and health extension workers who received training from teams composed of pediatric haemato-oncologists (PHOs), haemato-oncology fellows, pediatric residents, nurses, a nutritionist, and a clinical psychologist. Those responsible for remote supervision were general practitioners, nurses, and health extension workers who had been trained by the mentors. We have clearly elaborated in method section line 204 to 212.***

* “Median practice proficiency increased to 100 at primary level and to 93.8 at secondary level.” – Why did you remove the percentage? What were the pre-intervention levels? It’s impossible to judge the impact of the intervention without knowing the pre-intervention levels (as done in the prior sentence).

***Response to Editors: Thank you very much for the comments. We have included both the pre and post test result.***

AUTHOR SUMMARY

* “Median provider knowledge scores increased to 90.9, and practice proficiency increased to 100 at primary level and 93.8 at secondary level.” – Similar to our comment above, we ask you to include both pre- and post-intervention values.

***Response to Editors: Thank you very much for the comments. We have included both the pre and post result.***

INTRODUCTION
* Based on the track changes and a comparison of the current and prior manuscript versions, it doesn't appear that you have streamlined the introduction. The introduction is still repetitive in some parts and needs to be streamlined. Please revise it accordingly.

***Response to Editors: Thank you very much for the comments. We have streamlined the introduction to improve clarity and readability.***

METHODS AND RESULTS

* Figure 1: In the rebuttal, you indicated that you translated the figure text. However, the translation appears to be missing.

***Response to editors: Thank you very much for your suggestions. After discussion, we removed the figure, as its absence does not affect the quality of the manuscript. It only showed the group exercise conducted in the local language (Amharic).***

* While Figure 2 depicts the type of delays, it does not contain any information on the intervention timeline, the assessment types (i.e., main outcome measure) and when these assessments took place. Please re-consider adjusting the figure.

***Response to editors: Thank you very much for the comments. We have clearly described the intervention timeline under the Methods section, specifically under the Study Intervention subsection. The figure illustrates how patient delay, diagnostic delay, and treatment delay were calculated.***

*"Two analysts independently coded transcripts,” – please provide the initials. Response to the editors: Thank you very much for the comments. We have included it in the abstract section." – Please note that we did not ask you to include this detail in the abstract. Rather, we asked you to mention the initials of the two analysts so that they can be identified in the author/acknowledgment list.

***Response to editors: Thank you very much for suggestions. Sorry for the miss understandings we have include the initials of the two analysts (AAT and ZA).***
* Table 1: The definition for the asterisk (Other*), ‘BSc’, and ‘MSc/MPH’ is missing below the table. Please add.

***Response to editors: Thank you very much for your valuable comments. We have included the definition of all.***

* Table 2: The definition for ‘Certificate’ and ‘first degree’ is missing below the table. Please add.

***Response to editors: Thank you very much for your valuable comments. We have included the definition of the certificate. Based on previous feedback, we replaced ‘first degree’ with ‘BSc’ and have clearly defined BSc in the manuscript.***

* Figure 3: Please add a unit for the y-axis in the graph.

***Response to Editors: Thank you very much for comments. The y axis refereeing the to the knowledge, attitude, and practice scores out of 100, we have indicated it in the figure description.***

* “Figure 3: Please ensure to provide the relevant data behind the graphs in a supplementary table.”  - You have stated that you have included the necessary data, but you don’t cite any supplementary table. Where can readers find the values behind the graph?

***Response to editors: Thank you very much for the comments. We have cited the S3 file as a supplementary information.***

* Table 3: When reporting a median, please include the interquartile range.

***Response to editors: Thank you very much for the comments. Thank you very much for the comments. We have included the interquartile range value.***

* “Seven hundred forty-seven (70 %) of participants reported that joint oncology case conferences…” – please ensure to provide numerator and denominator. Please revise throughout the entire manuscript.

***Response to editors: Thank you very much for the comments. We include the denominator values.***

DISCUSSION
* Please remove all subheadings from the discussion (Limitations, Conclusions).

***Response to editors: Thank you very much for the suggestion. We remove the subheadings.***
