# Supplementary material for: Multilevel onsite training and mentorship model to accelerate early childhood cancer diagnosis in Northwest Ethiopia: A quasi-experimental mixed method study
Source: PLoS Med. 2026 Jun 29;23(6):e1005132. doi: 10.1371/journal.pmed.1005132 (PMC13313369; doi:10.1371/journal.pmed.1005132)
Supplement: Attachment — Submitted filename: Response to the reviewers.docx [file pmed.1005132.s008.docx]

Response to the reviewers

Dear reviewers,

We would like to sincerely thank you very much for your time and invaluable comments and suggestions on our manuscript.

We have carefully considered all your suggestions and comments, have revised the manuscript accordingly. Below, we have attached the authors' response to the reviewers' comments and suggestions, indicated in *Italics*.

**Reviewer #1:**

This is a mixed-method study that examined the association of a multilevel onsite training and mentorship model with knowledge-attitude-practice and clinical outcomes for early childhood cancer in Northwest Ethiopia. I only reviewed the quantitative part. I have some suggestions:

1) In abstract please be consistent in how the results were being reported. At the moment some were in absolute terms ('54.6 to 90.9') and some were in relative terms. Ideally both absolute and relative changes should be reported.

***Response from the authors:*** *Thank you very much for the suggestions. We appreciate the reviewer’s input; however, due to the nature of the data, we are obliged to report both the absolute and relative changes.*

2) The conclusion have strong causal implication, e.g. 'A context-tailored, multilevel training and mentorship model significantly improved provider capacity and reduced diagnostic delays for childhood cancer in Northwest Ethiopia' which I'm uncertain it's warranted. There are no control/comparison group and there were no randomisation. I'd suggest to reword as 'A context-tailored, multilevel training and mentorship model was associated with improvement in provider capacity and reduced diagnostic delays for childhood cancer in Northwest Ethiopia'

***Response from the authors:*** *Thank you very much for the suggestions. We accept the reviewer’s suggestion and have corrected it accordingly.*

3) p10 Methods: How were the 100 paediatric oncology charts selected for the evaluation?

***Response from the authors:*** *Thank you very much for the comment. All available charts are included in this study*

4) p11 Operational definition: Why the calculation of knowledge normalised score involved subtraction of 4? was it the minimise score to be 4? why?

***Response from the authors:*** *Thank you very much for the comment. We subtract 4 when calculating the normalized knowledge score because each knowledge question has four options, and the minimum possible score was 4. Therefore, to normalize the score, we deduct 4.*

5) p12 analysis: why Wilcoxon rank-sum test used for interval changes? weren't interval changes also paired in nature?

***Response from the authors****: Thank you very much for the comments. Our data consist of pre- and post-intervention scores from the same participants, which are paired in nature. We used the Wilcoxon signed-rank test to compare the median knowledge, attitude, and practice scores before and after the intervention, as the data were continuous and not normally distributed. For this reason, we used the Wilcoxon signed-rank test.*

6) Table 3. Please include median difference and p-values in the table.

***Response from the authors:*** *Thank you very much for the comments. We have made corrections based on the reviewer’s suggestions.*

7) Figure 3 is a bit hard to follow. Wouldn't it be more easy to plot the difference?

***Response from the authors:*** *Thank you for the comment. We agree that the box plot may be challenging to interpret, which is why we have provided a detailed explanation in the text to help readers understand it easily.*

8) **p11 'Contextual** factors such as staff turnover, supply stock outs, and concurrent health campaigns were logged monthly through mentorship reports and incorporated into analysis as potential effect modifiers' - I'm not sure if this was reported?

***Response from the authors:*** *Thank you very much for the comment. We have removed it, as it was incorrectly included in the document.*

9) Limitation is very well written though the interpretation (e.g. in conclusions, discussions) do not reflect those. Please reword all causal implications (e.g. 'effect', 'impact', 'improved', 'changed').

***Response from the authors****: Thank you very much. We address the reviewer’s comment.*

**Reviewer #2:**

Q1: The authors used "Three-Delay" model (adapted for pediatric oncology) which posits that delays arise at (1) the decision to seek care, (2) reaching a facility. These are patient related issues. It will be of interest if the authors can describe how these two aspects of cause of delays were addressed in the methods section and in the results section. Similarly, more details on community engagement by 3rd level participants were carried out and how the successes were evaluated.

***Response from the Authors:*** *Thank you very much for this insightful comment. We appreciate the opportunity to clarify how the adapted Three-Delay Model was operationalized in both the methods and results sections.*

*In our study, the Three-Delay Model for pediatric oncology was applied as follows:*

a) *Decision to seek care (Patient delay):*

*We described in the methods section how patient delay was measured using caregiver recall of symptom onset and the time taken to seek the first medical consultation. The results section presents the quantified duration of these delays and the key determinants (e.g., symptom recognition, cultural factors, and perceived severity).*

b) *Reaching a facility (Diagnostic delay):*

*We outlined in the methods how diagnostic delay was defined as the time from first consultation to arrival at a facility capable of providing diagnostic evaluation. In the results, we detail the median duration of diagnostic delays and factors influencing referral pathways, transportation barriers, and service availability.*

c) *Receiving timely diagnosis once at a facility (Treatment delay):*

*The methods section explains how treatment delay was calculated from the time the child reached a diagnostic center to the initiation of appropriate therapy. The results section highlights the major contributors to delays, including resource constraints and workflow bottlenecks.*

*With regard to community engagement by third-level participants, we have expanded the methods section to describe the specific activities undertaken—including awareness campaigns, caregiver education, and collaboration with local community structures. In the results section, we also report how the success of these community engagement activities was evaluated, drawing on both quantitative indicators (e.g., increased early presentation) and qualitative feedback from participants.*

**Reviewer #3:**

The work presented from Northwest Ethiopia is an important contribution to the ongoing debate on strengthening childhood cancer control in resource-constrained settings. By targeting primary, secondary, and tertiary levels simultaneously, the intervention acknowledges the reality that delays in pediatric oncology are rarely confined to a single tier of the health system, but rather accumulate across provider capacity, referral coordination, and community awareness. The impressive gains in knowledge and practice, coupled with measurable reductions in patient-to-provider and diagnosis intervals, demonstrate that a context-tailored mentorship model can produce tangible improvements even in fragile environments. Few comments for the authors.

C1: The full policy potential of this initiative could be further amplified by situating the findings more explicitly within the WHO CureAll framework, which provides a unifying set of global benchmarks. The CureAll indicators, i.e., symptom-to-first contact within 30 days, confirmation of diagnosis within 30 days, and treatment initiation within 30 days, are increasingly used as reference standards for national cancer control planning. In the present study, some of these thresholds appear to have been reached even before the intervention, and they are full acknowledged - but the presentation of the results has still been provided as % changes, not necessarily and always put against the benchmarks. With the use of a standard, as it is the WHO reference, the results would consolidate the progress made, to align with the quality standards of childhood cancer care. Also, a clearer alignment of results with these benchmarks would greatly strengthen the international comparability of the work and underline its policy relevance.

**Response from the authors:** *Thank you very much for the comments. We report the results as percent change and p-values to demonstrate whether the intervention had an effect. As indicated in Table 3, the pre-intervention values were above the WHO thresholds, whereas post-intervention values fell within the WHO thresholds. Therefore, we report the percent change and p-values to show the differences due to the intervention and their statistical significance.*

C2: Equally, the authors report that they retrieved 100 pediatric medical records for pre- vs post- quasi-experimental comparisons, that is informative; still, it remains unclear how these records were selected. Without explicit clarification of sampling criteria and bias mitigation strategies, readers may find it difficult to interpret the robustness of the conclusions. It is encouraged for the authors to specify these methodological details, as they are crucial for policy-makers and implementers who may seek to draw lessons from this experience.

***Response from the authors:*** *Thank you very much for the comment. All available charts are included in this study*

C3: Another important element is sustainability. The improvements reported here are compelling, but single-program evaluations cannot substitute for a system-wide monitoring architecture. Embedding the measurement of diagnostic and treatment intervals into routine health information systems, with transparent accountability mechanisms, is essential if gains are to be tracked, consolidated, and scaled nationally. This is mentioned in the conclusions, but could be better articulated.

*Response from the authors: Thank you for the comment. We agree that sustainability is crucial and have revised the conclusion to emphasize the need for embedding measurement of diagnostic and treatment intervals into routine health information systems with transparent accountability to ensure gains are tracked and scaled nationally.*

C4: Finally, this paper resonates with broader debates on workforce capacity building in oncology, where evidence remains scarce from low-income settings. Previous syntheses have underscored deep gaps across the availability, accessibility, acceptability, and quality of cancer providers. By demonstrating the effectiveness of tier-specific curricula, longitudinal mentorship, and community linkages, the Gondar experience provides a rare, practice-based exemplar of how AAAQ principles can be operationalized for childhood cancer. For this reason, it holds not only local significance but also relevance for informing national strategies and contributing to global learning.

*Response from the authors: Thank you very much for this thoughtful comment. We agree that our findings contribute to the broader discourse on oncology workforce capacity in low-income settings.*

Reviewer #4:

This manuscript reports on findings following a project to reduce late presentation of childhood cancer cases in Ethiopia by training health workers from the community, primary, secondary and tertiary health facilities. These multi-tiered trainings were done onsite at the tertiary treatment center and subsequently the participants were mentored in their facilities using various strategies which were modified based on various environmental situations that occurred. It is reported as a mixed methods study. It is an interesting manuscript with a number of good practices as examples for other similar settings to take note of and incorporate where possible.

Main comment is that some of the results presented and discussed are different from that shown in table 3. Definitions of interval terms used should be clearer. The qualitative aspect was not presented at all. Other remarks have been included in comments.

General: Should not use personal pronouns such as 'we' so such sentences should be re-worded appropriately.

***Response from the authors:*** *Thank you very much for your comments. We greatly appreciate your feedback and have made the corrections accordingly.*

Introduction:
Page 4 - 'These baseline realities high child cancer burden ---' Is there evidence for this high burden and what is this comparative to?

***Response from the authors:*** *Thank you very much for the comments. The comparative findings have been cited and incorporated into the main manuscript.*

Page 5 paragraph 3 - Sentence correction is required 'In Africa, clinician and community training has improved cancer recognition workshops in Tanzania and Uganda cut diagnostic delays—'

***Response from the authors:*** *Thank you very much for the comments. It has been corrected accordingly.*

Page 6 - Delay 3 - diagnostic capacity, what interventions were instituted to improve on diagnostic capacity? Was it only to speed up interval time and not accuracy of pathological diagnosis?

***Response from the authors:*** *Thank you very much for the comment. To improve diagnostic capacity, both the accuracy of pathological diagnosis and the reduction of turnaround time are essential.*

Method:
Page 16 - section on evolution is an exact repeat of section on page 15 under multisite level training. Should delete the section on evolution but compare the last sentence with the last sentence under multisite level training to ensure the facts there are correct.

***Response from the authors:*** *Thank you very much for the comments. It has been corrected based on the reviewers’ suggestions.*

Table 3 - Interval description. Why is the time from first symptoms to diagnosis computed as diagnostic delay instead of time from first medical contact to diagnosis being computed as diagnostic delay?

***Response from the authors:*** *Thank you very much for the comment. Diagnostic delay is calculated from the first symptom to diagnosis, whereas the time from first medical contact to diagnosis is considered physician delay, not diagnostic delay.*

Results:
Page 17. Physician delays - stated as increased post intervention. How was physician delay computed (description)?

***Response from the authors:*** *Thank you very much for the comment. As described above, physician delay is calculated as the time from first medical contact to diagnosis.*

State that 'the interval from diagnosis to treatment initiation increased by 11.9 percent (to 15.9 days, p = 0.15).' However, it has also been stated that 'Treatment initiation at the oncology center accelerated from a median of 69.0 to 15.9 days.' These facts do not seem to support what is being presented here.

***Response from the authors:*** *Thank you very much for the comments. As shown, the median change appears substantial, but it is not statistically significant. Further investigation may be needed.*

Page 20. The statement that 'lengthening diagnostic delays (56.5 → 65.5 days) and physician delays (27.0 → 30.5 days) post-intervention.' Is somewhat confusing as this diagnostic delay is not as shown in Table 3. This was a mixed methods study so results should include the qualitative component, this is missing.

***Response from the authors:*** *Thank you very much for the comment. The key indicators are patient delay, diagnostic delay, and treatment delay. Due to a typographical error, we were unable to correct this in the draft proposal. We have now deleted it to avoid any inconsistency.*

Discussion:
Too many results are repeated unnecessarily here.

Page 22. 'Treatment initiation intervals increased by 11.9 percent, from 14.2 to 15.9 days. This is different from what has been presented in Table 3.

***Response from the authors****: Thank you very much for the comments. We apologize for the inconvenience; we have corrected it accordingly. This occurred due to a lack of proofreading of our final manuscript.*

*Abstract: Please structure your abstract using the PLOS Medicine headings (Background, Methods and Findings, Conclusions). Please combine the Methods and Findings sections into one section.

***Response from the authors:*** *Thank you very much for the comments we have corrected it accordingly.*
