# Supplementary material for: Multilevel onsite training and mentorship model to accelerate early childhood cancer diagnosis in Northwest Ethiopia: A quasi-experimental mixed method study
Source: PLoS Med. 2026 Jun 29;23(6):e1005132. doi: 10.1371/journal.pmed.1005132 (PMC13313369; doi:10.1371/journal.pmed.1005132)
Supplement: Attachment — Submitted filename: R2_ Authors Response.docx [file pmed.1005132.s009.docx]

**Response to the reviewers and Editors**

Dear reviewers and Editors,

We would like to sincerely thank you very much for your time and invaluable comments and suggestions on our manuscript. We have carefully considered all your suggestions and comments, have revised the manuscript accordingly. Below, we have attached the authors' response to the reviewers' and editors’ comments and suggestions, indicated in ***Bold Italics***. **NA refers to ‘Not Applicable’**, meaning that no direct response is required for the suggestions raised by the reviewers and editors.

**Comments from Reviewers**:

**Reviewer #1:** Thanks for responding to my comments but I'm not sure if the authors have carefully gone through the paper to remove all causal implications. For example, in the main text conclusions, the text still reads 'a contextualized, multilevel onsite training and mentorship model can substantially *enhance* early childhood cancer diagnosis'. Please go through the manuscript to remove any causal wordings.

***Response to the reviewer****:* ***Thank you for this important comment. We have carefully reviewed the manuscript and revised the language throughout to avoid causal implications. Causal terms e.g., due to, led to, resulted in, strengthened were replaced with non-causal wording such as was associated with, was observed alongside, and aimed to enhance etc.***

In addition, my original question 5 asked about the Wilcoxon rank-sum test for interval changes even though the authors referred to the signed-rank test for the pre- and post- data. Please clarify the analysis of the interval changes and why rank-sum test was used.

***Response to the reviewer: Thank you very much for the comments. We have corrected it, and we apologize for the typo error. We have used Wilcoxon signed rank test due to its paired nature.***

**Reviewer #2:** The authors responses to the first review comments are satisfactory. I have no other comments

Thank you

***Response to the reviewer: NA***

**Reviewer #3:** no more comments.

***Response to the reviewer: NA***

**Reviewer #4:** This is a second review of the corrected manuscript on findings following a project to reduce late presentation and reduce diagnostic delay of childhood cancer cases in Ethiopia by training health workers from the community, primary, secondary and tertiary health facilities. In my opinion, the authors have addressed the comments sent to them and the manuscript is much improved.

***Response to the reviewer: NA***

**Requests from Editors**

Please note that the previous decision letter contained a list of editorial requests, to which we usually require a point-by-point response. Since you did not provide this response, please go back to the original letter and consider these points when revising your manuscript. Additionally, we have repeated the study-type-specific requests at the end of this letter and ask that you address them now.

We also find that the manuscript could be streamlined, and the presentation of the results could be improved. Please ensure that, where relevant, you clearly provide the n-number of participants and avoid reporting causality. Also, please ensure that you clearly provide uncertainty estimates (SD, CI values, etc.).

**Response to the editors:**

GENERAL
* Please confirm that your title complies with to PLOS Medicine's style. Your title must be nondeclarative and not a question. It should begin with main concept if possible. "Effect of" should be used only if causality can be inferred, i.e., for an RCT. Please place the study design ("A randomized controlled trial," "A retrospective study," "A modelling study," etc.) in the subtitle (ie, after a colon).

***Response to the editors: Thank you very much for the comment. We have included the study design in page 1 line 2 (Quasi-experimental mixed method study)***

* Statistical reporting: Please revise throughout the manuscript, including tables and figures.
- Please report statistical information as follows to improve clarity for the reader, "XX% (95% CI [XX, YY]; p</=)".

***Response to the editors: Thank you very much for the comments. We have revised the manuscript accordingly.***

- Please separate upper and lower bounds with commas instead of hyphens as the latter can be confused with reporting of negative values.

***Response to the editors: Thank you very much for the comments. We have revised the manuscript accordingly.***

- Please repeat statistical definitions (HR, CI etc.) for each set of parentheses.

***Response to the editors: Thank you very much for the comments. We have corrected the manuscript accordingly.***

* Please ensure that all abbreviations are defined at first use throughout the text (including statistical abbreviations).

***Response to the editors: Thank you very much for the comments. We have corrected the manuscript accordingly.***

* Please ensure that tables and figures, including those in supplementary files, are appropriately referenced in the main text.

***Response to the editors: Thank you very much for the comments. We have corrected the manuscript accordingly.***

* Please review your text for claims of novelty or primacy (e.g. 'for the first time' or ‘novel’) and remove this language.

***Response to the editors: Thank you very much for the comment. We remove terms like for the first time or novel in our manuscript.***

* Please confirm that any use of statistical terms (such as trend or significant) are supported by the data, and if not please remove them. The term trend should be used only when the test for trend has been conducted.

***Response to the editors: Thank you very much for the comments. We removed terms like trend and significant where we used other than statistical test.***

* Please provide titles and legends for all figures and tables (including those in Supporting Information files).

***Response to the editors: Thank you very much for the comments. We provide title and legends for all figures, tables, and supporting information files.***

* Please define all acronyms used in each figure or table in the corresponding legend.

***Response to the editors: Thank you very much for the comments. Thank you very much for the comments. We have corrected the manuscript accordingly.***

* Please confirm that you used patient-centered language. Please note that patient-centered language is constructed with the use of post-modified nouns putting the person first in the sentence structure.

***Response to the editors: Thank you very much for the comments. We have revised the manuscript accordingly.***

*  Please review your manuscript and edit to ensure compliance with our inclusive language requirements (<https://journals.plos.org/plosmedicine/s/human-subjects-research#loc-categorization>).
* The Data Availability Statement (DAS) in the online submission form requires revision. For each data source used in your study:

a) If the data are freely or publicly available, note this and state the location of the data: within the paper, in Supporting Information files, or in a public repository (include the DOI or accession number).

***Response to the editors: Thank you very much for the suggestions. Thank you very much for the comments. We have corrected the manuscript accordingly.***

b) If the data are owned by a third party but freely available upon request, please note this and state the owner of the data set and contact information for data requests (web or email address). Note that a study author cannot be the contact person for the data.

***Response to the editors: Thank you for the clarification. We confirm that the data are owned by a third party but are freely available upon request. We have provided the owner of the dataset and the appropriate contact information for data requests.***

c) If the data are not freely available, please describe briefly the ethical, legal, or contractual restriction that prevents you from sharing it. Please also include an appropriate contact (web or email address) for inquiries (again, this cannot be a study author).

***Response to the editors: NA***

* Please include the ‘Ethics approval and consent to participate’ statement in the Methods section of the main text.

***Response to the editors: Thank you very much for the comments. We have included it the method sections.***

* Please consider including an acknowledgment of study participants and of individuals who played a role in data collection or participant care.

***Response to the editors: Thank you very much for the comments. We have included it.***

* Please ensure to cite the Research implementation protocol in the Methods section of the main text.

***Response to the editors: Thank you very much for the comments. We have cited it properly.***

ABSTRACT
* Please confirm that your abstract complies with our requirements, including providing all the information relevant to this study type

<https://journals.plos.org/plosmedicine/s/submission-guidelines#loc-abstract>

***Response to the editors:*** ***Thank you for your comments. To the best of our knowledge, we have carefully revised the abstract to ensure that it complies with the journal’s requirements.***

* Please confirm that all numbers presented in the abstract are present and identical to numbers presented in the main manuscript text.

***Response to the editors: Thank you very much for the comments. We have verified that all numerical values in the abstract are accurately reflected and identical to those in the main manuscript.***

* In the last sentence of the Abstract Methods and Findings section, please describe the main limitation(s) of the study's methodology.

***Response to the editors: Thank you very much for the comments. We added that the main limitations of our study.***

* “three tiers of care” – please define.

***Response to the editors: Thank you very much for the comments. We include the definition at the method section.***

***Three tiers of care:*** ***In Ethiopia, the three tiers of care mean a structured health system with Primary (Health Posts, Health Centers, Primary Hospitals for basic/preventive care), Secondary (General Hospitals for broader services), and Tertiary (Specialized Hospitals for complex issues) levels, linked by a referral system for efficient, accessible care, with primary health care as the main entry point.***

* “primary-level and twenty-nine secondary-level” – what does primary-level and secondary-level refer to?

***Response to the editors: Thank you very much for the comments.***

***Primary Level (Basic & Preventive Care): refers to the first point of contact for most people, focusing on essential care, prevention, and basic treatment.***

***Secondary Level (Curative & Referral Care): Handles more complex problems and serves as the first referral point from primary care.***

***Tertiary Level (Specialized & Advanced Care): The highest level for very complex, specialized medical needs.***

* “over one thousand health extension workers” – can you provide the specific number?

***Response to the editors: Thank you very much for the comments. We have corrected it accordingly.***

* “A six-month mentorship program combined monthly onsite visits with remote supervision.” – Who were the mentors/mentees and who was responsible for the remote supervision?

***Response to the editors: Thank you very much for the comments. The mentors were pediatric haemato-oncologists (PHO),*** ***haemato-oncology fellows, pediatric residents, nurses, a nutritionist, and a clinical psychologist and those responsible for remote supervision were general practitioners, nurses, and health extension workers who had received training from the mentors.***

* “Knowledge-Attitude-Practice (KAP) surveys, patient-to-provider and diagnosis-to-treatment intervals, and qualitative interviews informed evaluation.” – What time points were covered? What were the main outcome measures?

***Response to the editors: Thank you very much for the comments.***

***Time points covered: Data collection occurred at clearly defined time points aligned with the intervention timeline. Quantitative and qualitative data were collected at baseline (pre-intervention) and endline (post-intervention). Baseline data were collected immediately prior to the initiation of the training and mentorship intervention to establish pre-existing levels of knowledge, attitudes, practices, and care-seeking timelines. Endline data were collected following completion of the intervention period to assess changes associated with the implementation of the program. For patient pathway indicators, routinely documented clinical records spanning the pre- and post-intervention periods were reviewed to capture changes over time in service delivery processes.***

***Main outcome measures: The evaluation was informed by three complementary data sources, each with predefined outcome measures:***

***Knowledge–Attitude–Practice (KAP) surveys: The primary quantitative outcomes included changes in healthcare providers’ knowledge, attitudes, and self-reported clinical practices related to childhood cancer recognition, referral, and early management. Knowledge outcomes assessed understanding of childhood cancer warning signs, referral criteria, and diagnostic pathways. Attitude outcomes captured perceptions of confidence, perceived importance of early diagnosis, and willingness to engage in referral and follow-up. Practice outcomes assessed reported changes in clinical behaviors, including screening, documentation, referral decision-making, and communication with caregivers. Pre- and post-intervention KAP scores were compared to evaluate improvements over time.***

***Patient-to-provider and diagnosis-to-treatment intervals: Health system performance outcomes included time-based indicators derived from patient records. These comprised (a) the patient-to-provider interval, defined as the time between initial symptom recognition by caregivers and first contact with a formal healthcare provider, and (b) the diagnosis-to-treatment interval, defined as the time between confirmed diagnosis and initiation of definitive treatment. Changes in these intervals between the pre- and post-intervention periods were used to assess whether the intervention was associated with improvements in timeliness of care and referral efficiency.***

***Qualitative interviews: Qualitative outcomes were derived from semi-structured interviews conducted with trained clinicians and mentors at endline. These interviews explored perceived changes in clinical competence, referral practices, interdisciplinary communication, and confidence in managing suspected childhood cancer cases. Additional outcomes included perceived facilitators and barriers to implementation, feasibility of remote mentorship, acceptability of the training model, and contextual factors influencing sustainability. Thematic analysis was used to identify recurring patterns and to triangulate findings from the quantitative components.***

* “Median knowledge scores”, “Median practice proficiency” – How were these assessed?

***Response to the editors: Thank you very much for the comments. Knowledge and practice were assessed using a standard questionnaire to determine whether participants had knowledge and whether they practiced it, supplemented by practical observations.***

* “Practice proficiency increased to 100 percent at primary sites and to 93.8 percent at secondary sites.” – compared to what percentage?

***Response to the editors: Thank you very much for the comments. As percentages are calculated out of 100, the pre–post values are 100% and 93.8%, representing the primary- and secondary-level health care tiers, respectively. We have corrected the term ‘site’ to ‘level’.***

* “A context-tailored, multilevel training and mentorship model significantly improved provider capacity…” – are you referring to statistical significance?

***Response to the editors: Thank you very much for your comments. Yes, we are referring to statistical significance.***

AUTHOR SUMMARY

* We ask that you include a short, non-technical Author Summary of your research to make findings accessible to a wide audience that includes both scientists and non-scientists. The **Author Summary** should immediately follow the Abstract in your revised manuscript. This text is subject to editorial change and should be distinct from the scientific abstract. Ideally each sub-heading should contain 2-3 single sentence, concise bullet points containing the most salient points from your study. In the final bullet point of 'What Do These Findings Mean?', please include the main limitations of the study in non-technical language. Please see our author guidelines for more

information: <https://journals.plos.org/plosmedicine/s/revising-your-manuscript#loc-author-summary>.

***Response to editors: Thank you very much for the comments. We have included the author summary in the revised manuscript.***
INTRODUCTION
* Please remove any subheadings.

***Response to the editors: Thank you very much for the comments. We have revised it accordingly.***

* We find that the introduction is repetitive in some parts, and it could be streamlined. Additionally, we suggest removing references to this manuscript as a “final report” when no other reports have been outlined.

***Response to the editors: Thank you very for the comments. We have corrected it accordingly.***

METHODS AND RESULTS

* Figure 1: We think that Figure 1 will only be useful for a broad audience if an English translation is provided.

***Response to the editors:*** ***Thank you very much for the comments. We have clearly translated at the figure descriptions. The figure in the Amharic language is provided as additional information and to illustrate the modality of the intervention at the community, facility, and health-care tiers.***

* We think a graph depicting the intervention timeline and assessment types and time points would be useful.

***Thank you very much for the comments. We have explained this in Figure 2. To avoid redundancy, we believe it is not necessary to present the same information in an additional graph, as the intervention modality is already described in the Methods section.***

* “Study of the Intervention” (page 8 ff) – The intervention, as described here, is the information we need you to include in the Abstract Methods.

***Response to the editors: Thank you very much for the comments. We have included it in the abstract section.***

* “Two analysts independently coded transcripts,” – please provide the initials.

***Response to the editors: Thank you very much for the comments. We have included it in the abstract section.***

* It is not necessary to describe the participants’ ages twice (directly under ‘Results’ and ‘Level 1/2 Participants’). Please revise.

***Response to the editors: Thank you very much for the comment. We have corrected it accordingly.***

* Please ensure to use a consistent abbreviation for ‘standard deviation’.

***Response to the editors: Thank you very much for the comments. We have corrected it accordingly.***

* The terms gender and sex are not interchangeable (as discussed in <https://www.who.int/health-topics/gender#tab=tab_1> ); please use the appropriate term.

***Response to the editors: Thank you very much for the comment. We have corrected it. As we are referring to biological differences, the appropriate term is sex.***

* Table 1/2: Was ‘Experience’ measured in years?

***Response to the editors: Thank you very much for the comment. We measured the experiences in years and indicated in the manuscript.***

* Table 1/2: We suggest changing ‘Training on childhood cancer’ to ‘Prior Training on childhood cancer’.

***Response to the editors: Thank you very much for the comments. We have revised it accordingly.***

* Table 1: Where is the definition for the asterisk (Other*)? What does first and second degree refer to?

***Response to the editors: Thank you very much for the comment. In the Ethiopian education system, a first degree refers to individuals who have completed Grade 12 and undertaken three to five years of training at public or private universities or colleges, resulting in a Bachelor of Science (BSc) or Bachelor of Arts (BA). A second degree refers to individuals who undertake specialized training in addition to their first degree, leading to a Master of Science (MSc), Master of Public Health (MPH), or Master of Arts (MA).***

* Table 2: How is ‘Certificate’ and ‘first degree’ defined?

***Response to the editors: Thank you very much for the comment. In the Ethiopian education system, a certificate refers to individuals who have completed Grade 10 and undertaken one-year training programs. A first degree refers*** ***to individuals who have completed Grade 12 and undertaken three to five years of training at public universities or colleges.***

* “Bad attitude scores declined modestly at both levels (Level I: –3.2; Level II: –6.7).” – We don't think "bad attitude" is an adequate description here. Please revise.

***Response to the editors: Thank you very much for the comments. We have revised it and replaced ‘bad’ with ‘******unfavorable’.***

* Figure 3: Please define all elements of box plots in the figure caption - center line, box limits and whiskers. Please add a unit for the y-axis and define all abbreviations used in the figure description.

***Response to the editors: Thank you very much for the comment.*** ***In the box plots, the center line represents the median; the lower and upper box limits indicate the first (Q1) and third (Q3) quartiles; and the whiskers extend to the minimum and maximum values. The y-axis refers to the percentage of knowledge, attitude, and practice.***

* Figure 3: Please ensure to provide the relevant data behind the graphs in a supplementary table.

***Response to the editors: Thank you very much for the comment. We have included the necessary data.***

* Table 3: When reporting a median, please include the interquartile range.

***Response to the editors: Thank you very much for the comments. We have included it.***

* “c. Contextual Interactions” – Please remove any numbering from headings/subheadings.

***Response to the editors: Thank you very much for the comments. We have corrected it accordingly.***

* “Facilities receiving intensified mentorship” – how many facilities received intensified mentorship?

***Response to the editors: Thank you very much for the comments.*** ***A total of 422 health facilities received mentorship (14 primary and general hospitals, 68 health centers, and 340 health posts).***

* “70 percent of participants” – please provide the total n-number.

***Response to the editors: Thank you very much for the comments. We have corrected it accordingly.***

* “Several HEWs” – how many exactly?

***Response to the editors: Thank you very much for the comments. We have revised it accordingly.***

DISCUSSION
* Please remove the 'conclusions' subheading from the discussion. Please also remove any other subheadings from the discussion.

***Response to the editors: Thank you very much for the comments. According to the manuscript submission guidelines, the Discussion and Conclusion sections should be presented under separate headings, and we have done so accordingly.***

*” Next steps involve the planned final impact evaluation in mid-2025, dissemination of peer reviewed findings, and pilot scale up in neighboring regions under Ministry leadership.” –Since we are now in 2026, we believe this statement should be updated.

***Response to the editors: Thank you very much for the comments. We have revised it accordingly.***

PREVIOUS EDITORIAL REQUESTS

* Abstract: Please include the study design, population and setting, number of participants, years during which the study took place (enrollment and follow up), length of follow up, and main outcome measures.

***Response to the editors: Thank you very much for the comments. We correct it accordingly.***

* Please state in the Methods section whether the study had a prospective protocol or analysis plan. If a prospective analysis plan (from your funding proposal, IRB or other ethics committee submission, study protocol, or other planning document written before analyzing the data) was used in designing the study, please include the relevant document(s) with your revised manuscript as a Supporting Information file to be published alongside your study and cite it in the Methods section. A legend for this file should be included at the end of your manuscript. If no such document exists, please make sure that the Methods section transparently describes when analyses were planned, and when/why any data-driven changes to analyses took place. Changes in the analysis, including those made in response to peer review comments, should be identified as such in the Methods section of the paper, with rationale.

***Response to the editors: Thank you very much for the comments. We have included the research implementation protocol in the Methods section as a supplementary file.***

* Please report your qualitative study according to the appropriate study design provided at (<http://www.equator-network.org/?post_type=eq_guidelines&eq_guidelines_study_design=qualitative-research&eq_guidelines_clinical_specialty=0&eq_guidelines_report_section=0&s=>) and provide the relevant completed checklist as a supplemental file. In the checklist, please include sufficient text excerpted from the manuscript to explain how you accomplished all applicable items. When completing checklists, please use section and paragraph numbers, rather than page numbers.

***Response to the editors: Thank you very much for the comments. We have revised it accordingly.***

* We recommend that authors use the COREQ checklist, or other relevant checklists listed by the Equator Network, such as the SRQR, to ensure complete reporting (see: <http://www.equator-network.org/?post_type=eq_guidelines&eq_guidelines_study_design=qualitative-research&eq_guidelines_clinical_specialty=0&eq_guidelines_report_section=0&s=>). Please add the following statement, or similar, to the Methods: "This study is reported as per the Consolidated criteria for reporting qualitative research (COREQ): a 32-item checklist for interviews and focus groups (S1 Checklist)."

***Response to the editors: Thank you very much for the comments. We have included the COREQ checklist as S2 file.***

* In general, we expect qualitative studies to include the following:

1) defined objectives or research questions;

Response to the editors: Thank you very much for the comments. **The primary objective of *this study was to evaluate the effectiveness of a multilevel onsite training and mentorship model in accelerating early diagnosis of childhood cancer in Northwest Ethiopia.***

***The study addressed the following research questions:***

- ***Does a multilevel onsite training and mentorship intervention improve early recognition and referral of suspected childhood cancer cases?***
- ***What contextual and health-system factors influence the effectiveness of the intervention?***

2) description of the sampling strategy, including rationale for the recruitment method, participant inclusion/exclusion criteria and the number of participants recruited;

***Response to the editors: Thank you very much for the comments. We used purposive sampling to recruit healthcare providers, mentors/trainers, and facility managers with at least six months of experience from intervention health facilities across multiple levels of care, excluding purely administrative staff, and continued in-depth interviews and focus group discussions until data saturation was achieved.***

3) Detailed reporting of the data collection procedures;

***Response to the editors: Thank you very much for the comments. Data were collected through semi-structured in-depth interviews and focus group discussions using a pre-tested guide that explored experiences with the training and mentorship, diagnostic challenges, perceived practice changes, and system-level facilitators and barriers; sessions were conducted in private facility settings, lasted 45–70 minutes, were audio-recorded with consent, supplemented by field notes, conducted in Amharic, transcribed verbatim, and translated into English for analysis.***

4) Data analysis procedures described in sufficient detail to enable replication;

***Response to the editors: Thank you very much for the comments. We conducted a thematic analysis using an inductive–deductive approach, involving repeated transcript familiarization, development of codes from research questions and emergent patterns, synthesis of codes into categories and themes, independent coding by multiple researchers with discrepancies resolved through discussion, and the use of reflexive memos and iterative refinement to ensure analytic transparency.***

5) A discussion of potential sources of bias; and

***Response to the editors: Thank you very much for the comments. Potential sources of bias included social desirability bias, researcher bias due to familiarity with the intervention, and selection bias arising from purposive sampling of intervention participants; these were mitigated through neutral probing, triangulation of data sources, and consensus coding among researchers.***

6) A discussion of limitations.

***Response to the editors: Thank you very much for the comments. The findings may have limited transferability beyond the selected facilities in Northwest Ethiopia, rely on self-reported experiences that may not fully reflect actual behavioral changes in practice, and be subject to subtle loss of nuance due to translation from local languages despite careful verification.***

* For the survey-based part of your study: Please ensure that the study is reported according to the CROSS guideline (<https://www.equator-network.org/reporting-guidelines/a-consensus-based-checklist-for-reporting-of-survey-studies-cross/>) and include the completed CROSS checklist as Supporting Information. Please add the following statement, or similar, to the Methods: "This study is reported as per A Consensus-Based Checklist for Reporting of Survey Studies (CROSS) guideline (S1 Checklist)." When completing the checklist, please use section and paragraph numbers, rather than page numbers.

***Response to the editors: Thank you very much for the comments. Thank you for this suggestion. We have revised the Methods section to explicitly state that the survey-based component of the study is reported in accordance with the A Consensus-Based Checklist for Reporting of Survey Studies (CROSS) guideline. The following statement has been added to the Methods:***

“This study is reported as per the A Consensus-Based Checklist for Reporting of Survey Studies (CROSS) guideline (S2 file).”

In addition, a completed CROSS checklist has been included as Supporting Information (S2 File), with section and paragraph numbers provided as requested.

* Please report your survey response rates according to AAPOR recommendations (<https://aapor.org/standards-and-ethics/best-practices/>)

***Response to the editors: Thank you very much for the comments. We have revised the Methods section to report survey response rates in accordance with AAPOR best practice recommendations. The definitions and calculations used to derive response rates are now clearly described, and the final response rate is reported transparently.***

* Please define how the population surveyed was sampled.

***Response to the editors: Thank you very much for the comments. We have clarified the sampling strategy used for the survey in the Methods section. Specifically, we describe the target population, sampling frame, eligibility criteria, and the recruitment approach used to invite participants to the survey.***

* Please compare characteristics of respondents and nonrespondents if possible.

***Response to the editors: Thank you very much for the comments. Where data were available, we have added a comparison of key characteristics between survey respondents and nonrespondents in the Methods/Results section to assess potential nonresponse bias. Where such comparisons were not feasible, this has been clearly acknowledged as a limitation.***

* If sequential waves of the survey were sent, please specify whether the characteristics of respondents changed over time or remained constant.

***Response to the editors: Thank you very much for the comments. We have clarified whether the survey was administered in sequential waves. Where applicable, we now report whether respondent characteristics remained consistent across waves or showed variation over time.***

* Please include the survey response rate in the Abstract.

***Response to the editors: Thank you very much for the comments. The survey response rate has now been added to the Abstract to improve transparency and reporting completeness.***

* Please include a copy of the survey in the supplementary files.

***Response to the editors: Thank you very much for the comments. A complete copy of the survey instrument has been added to the Supplementary Files for reference and transparency*.**

------------------------------------------------------------

General Editorial Requests

1) We ask every co-author listed on the manuscript to fill in a contributing author statement. If any of the co-authors have not filled in the statement, we will remind them to do so when the paper is revised. If all statements are not completed in a timely fashion this could hold up the re-review process. Should there be a problem getting one of your co-authors to fill in a statement we will be in contact. YOU MUST NOT ADD OR REMOVE AUTHORS UNLESS YOU HAVE ALERTED THE EDITOR HANDLING THE MANUSCRIPT TO THE CHANGE AND THEY SPECIFICALLY HAVE AGREED TO IT.

***Response to the editors: Thank you very for the comments. We strictly follow the journal guidelines and act accordingly.***

2) Please ensure that the paper adheres to the PLOS Data Availability Policy (see <http://journals.plos.org/plosmedicine/s/data-availability>), which requires that all data underlying the study's findings be provided in a repository or as Supporting Information. For data residing with a third party, authors are required to provide instructions with contact information for obtaining the data. PLOS journals do not allow statements supported by "data not shown" or "unpublished results." For such statements, authors must provide supporting data or cite public sources that include it.

***Response to the editors: Thank you very much for the comments. We have followed the journal guideline and act accordingly.***

3) Please review your reference list to ensure that it is complete and correct. If you have cited papers that have been retracted, please include the rationale for doing so in the manuscript text, or remove these references and replace them with relevant current references. Any changes to the reference list should be mentioned in the rebuttal letter that accompanies your revised manuscript.

***Response to the editors: Thank you very for the comments. However, we have not made any changes in the manuscript references.***
