# Supplementary material for: Multilevel onsite training and mentorship model to accelerate early childhood cancer diagnosis in Northwest Ethiopia: A quasi-experimental mixed method study
Source: PLoS Med. 2026 Jun 29;23(6):e1005132. doi: 10.1371/journal.pmed.1005132 (PMC13313369; doi:10.1371/journal.pmed.1005132)
Supplement: Attachment — Submitted filename: Abstract_PMEDICINE-D-25-02106R4.docx [file pmed.1005132.s011.docx]

**Abstract**

**Background**: Childhood cancer survival in low‐income countries remains below twenty percent, with late recognition and delayed referral commonly reported. In Northwest Ethiopia, care providers often lack the knowledge, skills, and support systems for recognizing early warning signs of pediatric malignancies. Community caregivers often present only after symptoms become advanced. The University of Gondar Comprehensive Specialized Hospital piloted a quality improvement initiative aimed at achieving earlier diagnoses. This study reports whether a multilevel onsite training and mentorship model could improve early recognition, referral practices, and timely diagnosis of childhood cancer in Northwest Ethiopia.

**Methods and findings:** From September 2024 through August 2025, a quasi-experimental pre-post, mixed-methods study design was employed across three tiers of care: primary (e.g. health centers), secondary (general hospitals), and tertiary (specialized hospitals). Eighteen primary-level and twenty-nine secondary-level clinicians completed intensive, on-site training (ten and seven days, respectively), while one thousand twenty health extension workers received pictorial outreach modules. A six-month mentorship program combined monthly onsite visits with remote supervision. Mentees included general practitioners, nurses, health officers, and health extension workers mentored by a multidisciplinary team that included, for example, pediatric hemato-oncologists. KAP surveys and patient-journey intervals from paediatric oncology charts were assessed at baseline and six months post-implementation, complemented by qualitative interviews and focus groups guided by the CFIR framework. Tier-specific curricula addressed clinical signs, referral protocols, and data documentation. Mentorship activities were reported alongside improved skill retention, facilitated rapid communication between facilities, and supported problem-solving of emergent challenges. Community engagement leveraged health extension workers’ home visits and partnerships with local religious institutions to support awareness activities.

In this study the participants had a 100% response rate. Median knowledge scores at primary and secondary levels rose from 54.6 to 90.9 and from 36.4 to 90.9, respectively. Median practice proficiency increased 87.5 to 100 at primary level and 68.8 to 93.8 at secondary level. The median interval from symptom onset to first health contact fell by 10.3 % (from 27.0 to 24.5 days), and the time from first contact to confirmed diagnosis decreased by 54.2 %. Treatment initiation interval increased by 11.9 %, reflecting ongoing infrastructural constraints. Qualitative findings underscored the roles of supportive leadership, diagnostic supply limitations, cultural beliefs, and referral coordination in shaping outcomes. This quasi-experimental pre-post design without a control group limits strong causal inference, especially in the presence of potential confounders like parallel public-health initiatives and seasonal variations in care-seeking.

**Conclusions:** A context-tailored, multilevel training and mentorship model was associated with improvement in provider capacity and reduced diagnostic delays for childhood cancer in Northwest Ethiopia. Further progress toward timely treatment may require investments in diagnostic infrastructure and referral system strengthening are required. The initiative’s tools and protocols are now positioned for national scale-up and integration into routine continuing medical education. The program’s high fidelity, adaptability in conflict zones, and alignment with Ministry of Health priorities are consistent with broader scale-up and integration into national continuing medical education frameworks.

**Keywords**: Childhood cancer, Early diagnosis, Multilevel Onsite training
