# Supplementary material for: Multilevel onsite training and mentorship model to accelerate early childhood cancer diagnosis in Northwest Ethiopia: A quasi-experimental mixed method study
Source: PLoS Med. 2026 Jun 29;23(6):e1005132. doi: 10.1371/journal.pmed.1005132 (PMC13313369; doi:10.1371/journal.pmed.1005132)
Supplement: Attachment — Submitted filename: Response to Editors_ R6.docx [file pmed.1005132.s012.docx]

**Response to Editors’**

Dear Editors’

We have carefully considered the editorial concerns regarding our reporting quality and have thoroughly overhauled the manuscript in this revision. The Abstract has been rewritten to strictly follow *PLOS Medicine’s* structure and length requirements. To maximize transparency, we have aligned our methodology and results with standard reporting guidelines. We also took this opportunity to resolve previous data inconsistencies. Finally, as requested, the full manuscript has been professionally copy-edited to ensure it meets the journal’s standards for clarity and precision.

**## Comments from editors**

Below are a few requests that have not been addressed satisfactorily:

* TITLE (request from previous decision letter): You have neither implemented this change nor provided a response in the rebuttal. Please change the title to (use the exact wording): “ Multilevel Onsite Training and Mentorship Model to Accelerate Early Childhood Cancer Diagnosis in Northwest Ethiopia: A Quasi-experimental mixed method study”

**Response: Thank you very much for the comments. We apologize for the inconvenience and have implemented the corrections based on the editor's suggestions. Line 1**

* ABSTRACT (request from previous decision letter): In our last decision letter, we provided a file containing a revised abstract that included the details we had requested previously. Please note that you did not address this point in your rebuttal, nor did you revise the abstract according to the suggestions provided in the attached file and the requests in previous decision letters.

**Response: Thank you for the comments. To the best of our knowledge, we have corrected the variables for which confidence intervals could be estimated. However, we were unable to address variables where estimation was not possible. We have made every effort to incorporate the editors’ suggestions.**

* TABLE 3: Thank you for your response. We note that several values are still reported in the format “27 ± 49.” The use of “±” conventionally indicates a mean with a measure of dispersion (e.g., standard deviation or standard error), and this notation is not appropriate for reporting medians with interquartile ranges. If the intent is to report median and interquartile range, please revise the presentation using standard formats (e.g., median (IQR) or median [Q1–Q3]) and remove the ± notation. Please revise accordingly throughout the manuscript to ensure clarity and consistency with standard statistical reporting conventions.

**Response: Thank you very much for the comments. We have corrected it through the document accordingly. Line 341 to 348**

* FIGURE 2: From your response, it is unclear which file contains the data behind the graph, and you have not included a reference to the data in the figure description of Figure 2.

**Response:** **We appreciate the feedback. The misplaced citation has been corrected, and the manuscript has been updated accordingly. Line 332**
