# Supplementary material for: Multilevel onsite training and mentorship model to accelerate early childhood cancer diagnosis in Northwest Ethiopia: A quasi-experimental mixed method study
Source: PLoS Med. 2026 Jun 29;23(6):e1005132. doi: 10.1371/journal.pmed.1005132 (PMC13313369; doi:10.1371/journal.pmed.1005132)
Supplement: Peer review history

# Editor report: Decision Letter 0

**DOI:** 10.1371/journal.pmed.1005132.r001

**Authors:** Alexandra Tosun

17 Jun 2025

Dear Dr Andualem,

Thank you for submitting your manuscript entitled "A Multilevel Onsite Training and Mentorship Model to Accelerate Early Childhood Cancer Diagnosis in Northwest Ethiopia" for consideration by PLOS Medicine.

Your manuscript has now been evaluated by the PLOS Medicine editorial staff and I am writing to let you know that we would like to send your submission out for external peer review.

However, before we can send your manuscript to reviewers, we need you to complete your submission by providing the metadata that is required for full assessment. To this end, please login to Editorial Manager where you will find the paper in the 'Submissions Needing Revisions' folder on your homepage. Please click 'Revise Submission' from the Action Links and complete all additional questions in the submission questionnaire.

For clinical studies, please upload a copy of your trial study protocol as a supporting information file. The study protocol should be the version submitted for approval to the institutional review board or ethics committee, should include any amendments to the study protocol, as well as the date of their approval by the institutional review or ethics committee. Please also detail any deviations from the study protocol in the Methods section of your manuscript. The editors will consider the protocol and study conduct prior to a final decision for external review.

Please re-submit your manuscript within two working days, i.e. by Jun 19 2025.

Login to Editorial Manager here: https://www.editorialmanager.com/pmedicine

Once your full submission is complete, your paper will undergo a series of checks in preparation for peer review. Once your manuscript has passed all checks it will be sent out for review.

Feel free to email me at atosun@plos.org or us at plosmedicine@plos.org if you have any queries relating to your submission.

Kind regards,

Alexandra Tosun, PhD

Senior Editor

PLOS Medicine

# Peer review: Decision Letter 1

**DOI:** 10.1371/journal.pmed.1005132.r002

**Authors:** Alexandra Tosun

4 Nov 2025

Dear Dr Andualem,

Many thanks for submitting your manuscript "A Multilevel Onsite Training and Mentorship Model to Accelerate Early Childhood Cancer Diagnosis in Northwest Ethiopia" (PMEDICINE-D-25-02106R1) to PLOS Medicine. The paper has been reviewed by subject experts and a statistician; their comments are included below and can also be accessed here: [LINK]

As you will see, the reviewers find that the study addresses an important topic. They have outlined several points of concern and made suggestions to strengthen the paper further. After discussing the paper with the editorial team, I'm pleased to invite you to revise the paper in response to the reviewers' comments. We plan to send the revised paper to some or all of the original reviewers, and we cannot provide any guarantees at this stage regarding publication.

In addition to these revisions, you may need to complete some formatting changes, which you will receive in a follow up email. A member of our team will be in touch with a set of requests shortly. If you do not receive a separate email within a few days, please assume that checks have been completed, and no additional changes are required.

When you upload your revision, please include a point-by-point response that addresses all of the reviewer and editorial points, indicating the changes made in the manuscript and either an excerpt of the revised text or the location (eg: page and line number) where each change can be found. Please also be sure to check the general editorial comments at the end of this letter and include these in your point-by-point response. When you resubmit your paper, please include a clean version of the paper as the main article file and a version with changes tracked as a marked-up manuscript. It may also be helpful to check the guidelines for revised papers at http://journals.plos.org/plosmedicine/s/revising-your-manuscript for any that apply to your paper.

We ask that you submit your revision by Nov 25 2025. However, if this deadline is not feasible, please contact me by email, and we can discuss a suitable alternative.

Don't hesitate to contact me directly with any questions (atosun@plos.org).

Best regards,

Alexandra

Alexandra Tosun, PhD

Senior Editor

PLOS Medicine

atosun@plos.org

-----------------------------------------------------------

Comments from the reviewers:

Reviewer #1: This is a mixed-method study that examined the association of a multilevel onsite training and mentorship model with knowledge-attitude-practice and clinical outcomes for early childhood cancer in Northwest Ethiopia. I only reviewed the quantitative part. I have some suggestions:

1. In abstract please be consistent in how the results were being reported. At the moment some were in absolute terms ('54.6 to 90.9') and some were in relative terms. Ideally both absolute and relative changes should be reported.

2. The conclusion have strong causal implication, e.g. 'A context-tailored, multilevel training and mentorship model significantly improved provider capacity and reduced diagnostic delays for childhood cancer in Northwest Ethiopia' which I'm uncertain it's warranted. There are no control/comparison group and there were no randomisation. I'd suggest to reword as 'A context-tailored, multilevel training and mentorship model was associated with improvement in provider capacity and reduced diagnostic delays for childhood cancer in Northwest Ethiopia'

3. p10 Methods: How were the 100 paediatric oncology charts selected for the evaluation?

4. p11 Operational definition: Why the calculation of knowledge normalised score involved subtraction of 4? was it the minimise score to be 4? why?

5. p12 analysis: why Wilcoxon rank-sum test used for interval changes? weren't interval changes also paired in nature?

6. Table 3. Please include median difference and p-values in the table.

7. Figure 3 is a bit hard to follow. Wouldn't it be more easy to plot the difference?

8. p11 'Contextual factors such as staff turnover, supply stock outs, and concurrent health campaigns were logged monthly through mentorship reports and incorporated into analysis as potential effect modifiers' - I'm not sure if this was reported?

9. Limitation is very well written though the interpretation (e.g. in conclusions, discussions) do not reflect those. Please reword all causal implications (e.g. 'effect', 'impact', 'improved', 'changed').

Reviewer #2: The authors used "Three-Delay" model (adapted for pediatric oncology) which posits that delays arise at (1) the decision to seek care, (2) reaching a facility' . These sre patient related issues. It will be of interest if the authors can describe how these two aspects of cause of delays were addressed in the methods section and in the results section. Similarly, more details on community engagement by 3rd level participants were carried out and how the successes were evaluated.

Reviewer #3: The work presented from Northwest Ethiopia is an important contribution to the ongoing debate on strengthening childhood cancer control in resource-constrained settings. By targeting primary, secondary, and tertiary levels simultaneously, the intervention acknowledges the reality that delays in pediatric oncology are rarely confined to a single tier of the health system, but rather accumulate across provider capacity, referral coordination, and community awareness. The impressive gains in knowledge and practice, coupled with measurable reductions in patient-to-provider and diagnosis intervals, demonstrate that a context-tailored mentorship model can produce tangible improvements even in fragile environments.

Few comments for the authors.

The full policy potential of this initiative could be further amplified by situating the findings more explicitly within the WHO CureAll framework, which provides a unifying set of global benchmarks. The CureAll indicators, i.e., symptom-to-first contact within 30 days, confirmation of diagnosis within 30 days, and treatment initiation within 30 days, are increasingly used as reference standards for national cancer control planning. In the present study, some of these thresholds appear to have been reached even before the intervention, and they are full acknowledged - but the presentation of the results has still been provided as % changes, not necessarily and always put against the benchmarks. With the use of a standard, as it is the WHO reference, the results would consolidate the progress made, to align with the quality standards of childhood cancer care. Also, a clearer alignment of results with these benchmarks would greatly strengthen the international comparability of the work and underline its policy relevance.

Equally, the authors report that they retrieved 100 pediatric medical records for pre- vs post- quasi-experimental comparisons, that is informative; still, it remains unclear how these records were selected. Without explicit clarification of sampling criteria and bias mitigation strategies, readers may find it difficult to interpret the robustness of the conclusions. It is encouraged for the authors to specify these methodological details, as they are crucial for policy-makers and implementers who may seek to draw lessons from this experience.

Another important element is sustainability. The improvements reported here are compelling, but single-program evaluations cannot substitute for a system-wide monitoring architecture. Embedding the measurement of diagnostic and treatment intervals into routine health information systems, with transparent accountability mechanisms, is essential if gains are to be tracked, consolidated, and scaled nationally. This is mentioned in the conclusions, but could be better articulated.

Finally, this paper resonates with broader debates on workforce capacity building in oncology, where evidence remains scarce from low-income settings. Previous syntheses have underscored deep gaps across the availability, accessibility, acceptability, and quality of cancer providers. By demonstrating the effectiveness of tier-specific curricula, longitudinal mentorship, and community linkages, the Gondar experience provides a rare, practice-based exemplar of how AAAQ principles can be operationalised for childhood cancer. For this reason, it holds not only local significance but also relevance for informing national strategies and contributing to global learning.

Reviewer #4: This manuscript reports on findings following a project to reduce late presentation of childhood cancer cases in Ethiopia by training health workers from the community, primary, secondary and tertiary health facilities. These multi-tiered trainings were done onsite at the tertiary treatment centre and subsequently the participants were mentored in their facilities using various strategies which were modified based on various environmental situations that occurred. It is reported as a mixed methods study. It is an interesting manuscript with a number of good practices as examples for other similar settings to take note of and incorporate where possible. Main comment is that some of the results presented and discussed are different from that shown in table 3. Definitions of interval terms used should be clearer. The qualitative aspect was not presented at all. Other remarks have been included in comments.

General: Should not use personal pronouns such as 'we' so such sentences should be re-worded appropriately.

Introduction:

Page 4 - 'These baseline realities high child cancer burden ---' Is there evidence for this high burden and what is this comparative to?

Page 5 paragraph 3 - Sentence correction is required 'In Africa, clinician and community training has improved cancer recognition workshops in Tanzania and Uganda cut diagnostic delays—'

Page 6 - Delay 3 - diagnostic capacity, what interventions were instituted to improve on diagnostic capacity? Was it only to speed up interval time and not accuracy of pathological diagnosis?

Method:

Page 16 - section on evolution is an exact repeat of section on page 15 under multisite level training. Should delete the section on evolution but compare the last sentence with the last sentence under multisite level training to ensure the facts there are correct.

Table 3 - Interval description. Why is the time from first symptoms to diagnosis computed as diagnostic delay instead of time from first medical contact to diagnosis being computed as diagnostic delay?

Results:

Page 17. Physician delays - stated as increased post intervention. How was physician delay computed (description)?

State that 'the interval from diagnosis to treatment initiation increased by 11.9 percent (to 15.9 days, p = 0.15).' However, it has also been stated that 'Treatment initiation at the oncology center accelerated from a median of 69.0 to 15.9 days.' These facts do not seem to support what is being presented here.

Page 20. The statement that 'lengthening diagnostic delays (56.5 → 65.5 days) and physician delays (27.0 → 30.5 days) post-intervention.' Is somewhat confusing as this diagnostic delay is not as shown in Table 3.

This was a mixed methods study so results should include the qualitative component, this is missing.

Discussion:

Too many results are repeated unnecessarily here.

Page 22. 'treatment initiation intervals increased by 11.9 percent, from 14.2 to 15.9 days. This is different from what has been presented in Table 3.

Any attachments provided with reviews can be seen via the following link: [LINK]

--------------------------------------------------------- ---

General editorial requests:

(Note: not all will apply to your paper, but please check each item carefully)

*We ask every co-author listed on the manuscript to fill in a contributing author statement, making sure to declare all competing interests. If any of the co-authors have not filled in the statement, we will remind them to do so when the paper is revised. If all statements are not completed in a timely fashion this could hold up the re-review process. If new competing interests are declared later in the revision process, this may also hold up the submission. Should there be a problem getting one of your co-authors to fill in a statement we will be in contact. Please do not add or remove authors without first discussing this with the handling editor. You can see our competing interests policy here: http://journals.plos.org/plosmedicine/s/competing-interests.

*Please upload any figures associated with your paper as individual TIF or EPS files with 300dpi resolution at resubmission; please read our figure guidelines for more information on our requirements: http://journals.plos.org/plosmedicine/s/figures. While revising your submission, we strongly recommend that you use PLOS's NAAS tool (https://ngplosjournals.pagemajik.ai/artanalysis) to test your figure files. NAAS can convert your figure files to the TIFF file type and meet basic requirements (such as print size, resolution), or provide you with a report on issues that do not meet our requirements and that NAAS cannot fix.

After uploading your figures to PLOS's NAAS tool - https://ngplosjournals.pagemajik.ai/artanalysis, NAAS will process the files provided and display the results in the "Uploaded Files" section of the page as the processing is complete.

If the uploaded figures meet our requirements (or NAAS is able to fix the files to meet our requirements), the figure will be marked as "fixed" above. If NAAS is unable to fix the files, a red "failed" label will appear above.

When NAAS has confirmed that the figure files meet our requirements, please download the file via the download option, and include these NAAS processed figure files when submitting your revised manuscript.

*Please ensure that the paper adheres to the PLOS Data Availability Policy (see http://journals.plos.org/plosmedicine/s/data-availability), which requires that all data underlying the study's findings be provided in a repository or as Supporting Information. For data residing with a third party, authors are required to provide instructions with contact information (web or email address) for obtaining the data. Please note that a study author cannot be the contact person for the data. PLOS journals do not allow statements supported by "data not shown" or "unpublished results." For such statements, authors must provide supporting data or cite public sources that include it.

*We expect all researchers with submissions to PLOS in which author-generated code underpins the findings in the manuscript to make all author-generated code available without restrictions upon publication of the work. In cases where code is central to the manuscript, we may require the code to be made available as a condition of publication. Authors are responsible for ensuring that the code is reusable and well documented. Please make any custom code available, either as part of your data deposition or as a supplementary file. Please add a sentence to your data availability statement regarding any code used in the study, e.g. "The code used in the analysis is available from Github [URL] and archived in Zenodo [DOI link]" Please review our guidelines at https://journals.plos.org/plosmedicine/s/materials-software-and-code-sharing and ensure that your code is shared in a way that follows best practice and facilitates reproducibility and reuse. Because Github depositions can be readily changed or deleted, we encourage you to make a permanent DOI'd copy (e.g. in Zenodo) and provide the URL.

FORMATTING - GENERAL

*Abstract: Please structure your abstract using the PLOS Medicine headings (Background, Methods and Findings, Conclusions). Please combine the Methods and Findings sections into one section.

*At this stage, we ask that you include a short, non-technical Author Summary of your research to make findings accessible to a wide audience that includes both scientists and non-scientists. The Author Summary should immediately follow the Abstract in your revised manuscript. This text is subject to editorial change and should be distinct from the scientific abstract. Ideally each sub-heading should contain 2-3 single sentence, concise bullet points containing the most salient points from your study. In the final bullet point of 'What Do These Findings Mean?', please include the main limitations of the study in non-technical language. Please see our author guidelines for more information: https://journals.plos.org/plosmedicine/s/revising-your-manuscript#loc-author-summary.

*Please express the main results with 95% CIs as well as p values. When reporting p values please report as p<0.001 and where higher as the exact p value p=0.002, for example. Throughout, suggest reporting statistical information as follows to improve clarity for the reader "22% (95% CI [13%,28%]; p</=)". Please be sure to define all numerical values at first use.

*Please include page numbers and line numbers in the manuscript file. Use continuous line numbers (do not restart the numbering on each page).

*Please cite the reference numbers in square brackets. Citations should precede punctuation.

FIGURES AND TABLES

*Please provide titles and legends for all figures and tables (including those in Supporting Information files).

*Please define all abbreviations used in each figure/table (including those in Supporting Information files).

*Please consider avoiding the use of red and green in order to make your figure more accessible to those with color blindness.

SUPPLEMENTARY MATERIAL

*Please note that supplementary material will be posted as supplied by the authors. Therefore, please amend it according to the relevant comments outlined here.

*Please cite your Supporting Information as outlined here: https://journals.plos.org/plosmedicine/s/supporting-information

REFERENCES

*PLOS uses the numbered citation (citation-sequence) method and first six authors, et al.

*Please ensure that journal name abbreviations match those found in the National Center for Biotechnology Information (NCBI) databases (http://www.ncbi.nlm.nih.gov/nlmcatalog/journals), and are appropriately formatted and capitalised.

*Where website addresses are cited, please include the complete URL and specify the date of access (e.g. [accessed: 12/06/2023]).

*Please also see https://journals.plos.org/plosmedicine/s/submission-guidelines#loc-references for further details on reference formatting.

STUDY TYPE-SPECIFIC REQUESTS

*Abstract: Please include the study design, population and setting, number of participants, years during which the study took place (enrollment and follow up), length of follow up, and main outcome measures.

*Please state in the Methods section whether the study had a prospective protocol or analysis plan. If a prospective analysis plan (from your funding proposal, IRB or other ethics committee submission, study protocol, or other planning document written before analyzing the data) was used in designing the study, please include the relevant document(s) with your revised manuscript as a Supporting Information file to be published alongside your study and cite it in the Methods section. A legend for this file should be included at the end of your manuscript. If no such document exists, please make sure that the Methods section transparently describes when analyses were planned, and when/why any data-driven changes to analyses took place. Changes in the analysis, including those made in response to peer review comments, should be identified as such in the Methods section of the paper, with rationale.

*Please report your qualitative study according to the appropriate study design provided at (http://www.equator-network.org/?post_type=eq_guidelines&eq_guidelines_study_design=qualitative-research&eq_guidelines_clinical_specialty=0&eq_guidelines_report_section=0&s=) and provide the relevant completed checklist as a supplemental file. In the checklist, please include sufficient text excerpted from the manuscript to explain how you accomplished all applicable items. When completing checklists, please use section and paragraph numbers, rather than page numbers.

*We recommend that authors use the COREQ checklist, or other relevant checklists listed by the Equator Network, such as the SRQR, to ensure complete reporting (see: http://www.equator-network.org/?post_type=eq_guidelines&eq_guidelines_study_design=qualitative-research&eq_guidelines_clinical_specialty=0&eq_guidelines_report_section=0&s=). Please add the following statement, or similar, to the Methods: "This study is reported as per the Consolidated criteria for reporting qualitative research (COREQ): a 32-item checklist for interviews and focus groups (S1 Checklist)."

*In general, we expect qualitative studies to include the following: 1) defined objectives or research questions; 2) description of the sampling strategy, including rationale for the recruitment method, participant inclusion/exclusion criteria and the number of participants recruited; 3) detailed reporting of the data collection procedures; 4) data analysis procedures described in sufficient detail to enable replication; 5) a discussion of potential sources of bias; and 6) a discussion of limitations.

*For the survey-based part of your study: Please ensure that the study is reported according to the CROSS guideline (https://www.equator-network.org/reporting-guidelines/a-consensus-based-checklist-for-reporting-of-survey-studies-cross/) and include the completed CROSS checklist as Supporting Information. Please add the following statement, or similar, to the Methods: "This study is reported as per A Consensus-Based Checklist for Reporting of Survey Studies (CROSS) guideline (S1 Checklist)." When completing the checklist, please use section and paragraph numbers, rather than page numbers.

*Please report your survey response rates according to AAPOR recommendations (https://aapor.org/standards-and-ethics/best-practices/)

*Please define how the population surveyed was sampled.

*Please compare characteristics of respondents and nonrespondents if possible.

*If sequential waves of the survey were sent, please specify whether the characteristics of respondents changed over time or remained constant.

*Please include the survey response rate in the Abstract.

*Please include a copy of the survey in the supplementary files.

# Author comment: Author response to Decision Letter 2

**DOI:** 10.1371/journal.pmed.1005132.r003

1 Dec 2025

Attachment Submitted filename: Response to the reviewers.docx

# Peer review: Decision Letter 2

**DOI:** 10.1371/journal.pmed.1005132.r004

**Authors:** Alexandra Tosun

12 Jan 2026

Dear Dr. Andualem,

Thank you very much for re-submitting your manuscript "A Multilevel Onsite Training and Mentorship Model to Accelerate Early Childhood Cancer Diagnosis in Northwest Ethiopia" (PMEDICINE-D-25-02106R2) for review by PLOS Medicine.

Thank you for your detailed response to the reviewers' and editors’ comments. I have discussed the paper with my colleagues, and it has also been seen again by all of the original reviewers. The changes made to the paper were mostly satisfactory to the reviewers. As such, we intend to accept the paper for publication, pending your attention to the reviewers' and editors' comments below in a further revision. When submitting your revised paper, please once again include a detailed point-by-point response to the editorial comments. The remaining issues that need to be addressed are listed at the end of this email.

In revising the manuscript for further consideration here, please ensure you address the specific points made by each reviewer and the editors. In your rebuttal letter you should indicate your response to the reviewers' and editors' comments and the changes you have made in the manuscript. Please submit a clean version of the paper as the main article file. A version with changes marked must also be uploaded as a marked up manuscript file. Please also check the guidelines for revised papers at http://journals.plos.org/plosmedicine/s/revising-your-manuscript for any that apply to your paper. If you haven't already, we ask that you provide a short, non-technical Author Summary of your research to make findings accessible to a wide audience that includes both scientists and non-scientists. The Author Summary should immediately follow the Abstract in your revised manuscript. This text is subject to editorial change and should be distinct from the scientific abstract.

In addition to these revisions, you may need to complete some formatting changes, which you will receive in a follow up email. A member of our team will be in touch with a set of requests shortly. If you do not receive a separate email within a few days, please assume that checks have been completed, and no additional changes are required.

Please note, when your manuscript is accepted, an uncorrected proof of your manuscript will be published online ahead of the final version, unless you've already opted out via the online submission form. If, for any reason, you do not want an earlier version of your manuscript published online or are unsure if you have already indicated as such, please let the journal staff know immediately at plosmedicine@plos.org.

We ask that you submit your revision by Jan 19 2026. However, if this deadline is not feasible, please contact me (atosun@plos.org) or the journal staff by email, and we can discuss a suitable alternative.

We look forward to receiving the revised manuscript.

Sincerely,

Alexandra Tosun, PhD

Senior Editor

PLOS Medicine

plosmedicine.org

***Please note while forming your response, if your article is accepted, you may have the opportunity to make the peer review history publicly available. The record will include editor decision letters (with reviews) and your responses to reviewer comments. If eligible, we will contact you to opt in or out.***

------------------------------------------------------------

Comments from Reviewers:

Reviewer #1: Thanks for responding to my comments but I'm not sure if the authors have carefully gone through the paper to remove all causal implications. For example in the main text conclusions, the text still reads 'a contextualized, multilevel onsite training and mentorship model can substantially *enhance* early childhood cancer diagnosis'. Please go through the manuscript to remove any causal wordings.

In addition, my original question 5 asked about the Wilcoxon rank-sum test for interval changes even though the authors referred to the signed-rank test for the pre- and post- data. Please clarify the analysis of the interval changes and why rank-sum test was used.

Reviewer #2: The authors responses to the first review comments are satisfactory . I have no other comments

Thank you

Reviewer #3: no more comments.

Reviewer #4: This is a second review of the corrected manuscript on findings following a project to reduce late presentation and reduce diagnostic delay of childhood cancer cases in Ethiopia by training health workers from the community, primary, secondary and tertiary health facilities.

In my opinion, the authors have addressed the comments sent to them and the manuscript is much improved.

------------------------------------------------------------

Requests from Editors:

Please note that the previous decision letter contained a list of editorial requests, to which we usually require a point-by-point response. Since you did not provide this response, please go back to the original letter and consider these points when revising your manuscript. Additionally, we have repeated the study-type-specific requests at the end of this letter and ask that you address them now.

We also find that the manuscript could be streamlined, and the presentation of the results could be improved. Please ensure that, where relevant, you clearly provide the n-number of participants and avoid reporting causality. Also, please ensure that you clearly provide uncertainty estimates (SD, CI values, etc.).

GENERAL

* Please confirm that your title complies with to PLOS Medicine's style. Your title must be nondeclarative and not a question. It should begin with main concept if possible. "Effect of" should be used only if causality can be inferred, i.e., for an RCT. Please place the study design ("A randomized controlled trial," "A retrospective study," "A modelling study," etc.) in the subtitle (ie, after a colon).

* Statistical reporting: Please revise throughout the manuscript, including tables and figures.

- Please report statistical information as follows to improve clarity for the reader, ""XX% (95% CI [XX,YY]; p</=)"".

- Please separate upper and lower bounds with commas instead of hyphens as the latter can be confused with reporting of negative values.

- Please repeat statistical definitions (HR, CI etc.) for each set of parentheses.

* Please ensure that all abbreviations are defined at first use throughout the text (including statistical abbreviations).

* Please ensure that tables and figures, including those in supplementary files, are appropriately referenced in the main text.

* Please review your text for claims of novelty or primacy (e.g. 'for the first time' or ‘novel’) and remove this language.

* Please confirm that any use of statistical terms (such as trend or significant) are supported by the data, and if not please remove them. The term trend should be used only when the test for trend has been conducted.

* Please provide titles and legends for all figures and tables (including those in Supporting Information files).

* Please define all acronyms used in each figure or table in the corresponding legend.

* Please confirm that you used patient-centered language. Please note that patient-centered language is constructed with the use of post-modified nouns putting the person first in the sentence structure.

* Please review your manuscript and edit to ensure compliance with our inclusive language requirements (https://journals.plos.org/plosmedicine/s/human-subjects-research#loc-categorization).

* The Data Availability Statement (DAS) in the online submission form requires revision. For each data source used in your study:

a) If the data are freely or publicly available, note this and state the location of the data: within the paper, in Supporting Information files, or in a public repository (include the DOI or accession number).

b) If the data are owned by a third party but freely available upon request, please note this and state the owner of the data set and contact information for data requests (web or email address). Note that a study author cannot be the contact person for the data.

c) If the data are not freely available, please describe briefly the ethical, legal, or contractual restriction that prevents you from sharing it. Please also include an appropriate contact (web or email address) for inquiries (again, this cannot be a study author).

* Please include the ‘Ethics approval and consent to participate’ statement in the Methods section of the main text.

* Please consider including an acknowledgment of study participants and of individuals who played a role in data collection or participant care.

* Please ensure to cite the Research implementation protocol in the Methods section of the main text.

ABSTRACT

* Please confirm that your abstract complies with our requirements, including providing all the information relevant to this study type https://journals.plos.org/plosmedicine/s/submission-guidelines#loc-abstract

* Please confirm that all numbers presented in the abstract are present and identical to numbers presented in the main manuscript text.

* In the last sentence of the Abstract Methods and Findings section, please describe the main limitation(s) of the study's methodology.

* “three tiers of care” – please define.

* “primary-level and twenty-nine secondary-level” – what does primary-level and secondary-level refer to?

* “over one thousand health extension workers” – can you provide the specific number?

* “A six-month mentorship program combined monthly onsite visits with remote supervision.” – Who were the mentors/mentees and who was responsible for the remote supervision?

* “Knowledge-Attitude-Practice (KAP) surveys, patient-to-provider and diagnosis-to-treatment intervals, and qualitative interviews informed evaluation.” – What time points were covered? What were the main outcome measures?

* “Median knowledge scores”, “Median practice proficiency” – How were these assessed?

* “Practice proficiency increased to 100 percent at primary sites and to 93.8 percent at secondary sites.” – compared to what percentage?

* “A context-tailored, multilevel training and mentorship model significantly improved provider capacity…” – are you referring to statistical significance?

AUTHOR SUMMARY

* We ask that you include a short, non-technical Author Summary of your research to make findings accessible to a wide audience that includes both scientists and non-scientists. The Author Summary should immediately follow the Abstract in your revised manuscript. This text is subject to editorial change and should be distinct from the scientific abstract. Ideally each sub-heading should contain 2-3 single sentence, concise bullet points containing the most salient points from your study. In the final bullet point of 'What Do These Findings Mean?', please include the main limitations of the study in non-technical language. Please see our author guidelines for more information: https://journals.plos.org/plosmedicine/s/revising-your-manuscript#loc-author-summary.

INTRODUCTION

* Please remove any subheadings.

* We find that the introduction is repetitive in some parts, and it could be streamlined. Additionally, we suggest removing references to this manuscript as a “final report” when no other reports have been outlined.

METHODS AND RESULTS

* Figure 1: We think that Figure 1 will only be useful for a broad audience if an English translation is provided.

* We think a graph depicting the intervention timeline and assessment types and time points would be useful.

* “Study of the Intervention” (page 8 ff) – The intervention, as described here, is the information we need you to include in the Abstract Methods.

* “Two analysts independently coded transcripts,..” – please provide the initials.

* It is not necessary to describe the participants’ ages twice (directly under ‘Results’ and ‘Level 1/2 Participants’). Please revise.

* Please ensure to use a consistent abbreviation for ‘standard deviation’.

* The terms gender and sex are not interchangeable (as discussed in https://www.who.int/health-topics/gender#tab=tab_1 ); please use the appropriate term.

* Table 1/2: Was ‘Experience’ measured in years?

* Table 1/2: We suggest changing ‘Training on childhood cancer’ to ‘Prior Training on childhood cancer’.

* Table 1: Where is the definition for the asterisk (Other*)? What does first and second degree refer to?

* Table 2: How is ‘Certificate’ and ‘first degree’ defined?

* “Bad attitude scores declined modestly at both levels (Level I: –3.2; Level II: –6.7).” – We don't think "bad attitude" is an adequate description here. Please revise.

* Figure 3: Please define all elements of box plots in the figure caption - center line, box limits and whiskers. Please add a unit for the y-axis and define all abbreviations used in the figure description.

* Figure 3: Please ensure to provide the relevant data behind the graphs in a supplementary table.

* Table 3: When reporting a median, please include the interquartile range.

* “c. Contextual Interactions” – Please remove any numbering from headings/subheadings.

* “Facilities receiving intensified mentorship” – how many facilities received intensified mentorship?

* “70 percent of participants” – please provide the total n-number.

* “Several HEWs” – how many exactly?

DISCUSSION

* Please remove the 'conclusions' subheading from the discussion. Please also remove any other subheadings from the discussion.

*” Next steps involve the planned final impact evaluation in mid-2025, dissemination of peer reviewed findings, and pilot scale up in neighbouring regions under Ministry leadership.” – Since we are now in 2026, we believe this statement should be updated.

PREVIOUS EDITORIAL REQUESTS

* Abstract: Please include the study design, population and setting, number of participants, years during which the study took place (enrollment and follow up), length of follow up, and main outcome measures.

* Please state in the Methods section whether the study had a prospective protocol or analysis plan. If a prospective analysis plan (from your funding proposal, IRB or other ethics committee submission, study protocol, or other planning document written before analyzing the data) was used in designing the study, please include the relevant document(s) with your revised manuscript as a Supporting Information file to be published alongside your study and cite it in the Methods section. A legend for this file should be included at the end of your manuscript. If no such document exists, please make sure that the Methods section transparently describes when analyses were planned, and when/why any data-driven changes to analyses took place. Changes in the analysis, including those made in response to peer review comments, should be identified as such in the Methods section of the paper, with rationale.

* Please report your qualitative study according to the appropriate study design provided at (http://www.equator-network.org/?post_type=eq_guidelines&eq_guidelines_study_design=qualitative-research&eq_guidelines_clinical_specialty=0&eq_guidelines_report_section=0&s=) and provide the relevant completed checklist as a supplemental file. In the checklist, please include sufficient text excerpted from the manuscript to explain how you accomplished all applicable items. When completing checklists, please use section and paragraph numbers, rather than page numbers.

* We recommend that authors use the COREQ checklist, or other relevant checklists listed by the Equator Network, such as the SRQR, to ensure complete reporting (see: http://www.equator-network.org/?post_type=eq_guidelines&eq_guidelines_study_design=qualitative-research&eq_guidelines_clinical_specialty=0&eq_guidelines_report_section=0&s=). Please add the following statement, or similar, to the Methods: "This study is reported as per the Consolidated criteria for reporting qualitative research (COREQ): a 32-item checklist for interviews and focus groups (S1 Checklist)."

* In general, we expect qualitative studies to include the following: 1) defined objectives or research questions; 2) description of the sampling strategy, including rationale for the recruitment method, participant inclusion/exclusion criteria and the number of participants recruited; 3) detailed reporting of the data collection procedures; 4) data analysis procedures described in sufficient detail to enable replication; 5) a discussion of potential sources of bias; and 6) a discussion of limitations.

* For the survey-based part of your study: Please ensure that the study is reported according to the CROSS guideline (https://www.equator-network.org/reporting-guidelines/a-consensus-based-checklist-for-reporting-of-survey-studies-cross/) and include the completed CROSS checklist as Supporting Information. Please add the following statement, or similar, to the Methods: "This study is reported as per A Consensus-Based Checklist for Reporting of Survey Studies (CROSS) guideline (S1 Checklist)." When completing the checklist, please use section and paragraph numbers, rather than page numbers.

* Please report your survey response rates according to AAPOR recommendations (https://aapor.org/standards-and-ethics/best-practices/)

* Please define how the population surveyed was sampled.

* Please compare characteristics of respondents and nonrespondents if possible.

* If sequential waves of the survey were sent, please specify whether the characteristics of respondents changed over time or remained constant.

* Please include the survey response rate in the Abstract.

* Please include a copy of the survey in the supplementary files.

------------------------------------------------------------

General Editorial Requests

1) We ask every co-author listed on the manuscript to fill in a contributing author statement. If any of the co-authors have not filled in the statement, we will remind them to do so when the paper is revised. If all statements are not completed in a timely fashion this could hold up the re-review process. Should there be a problem getting one of your co-authors to fill in a statement we will be in contact. YOU MUST NOT ADD OR REMOVE AUTHORS UNLESS YOU HAVE ALERTED THE EDITOR HANDLING THE MANUSCRIPT TO THE CHANGE AND THEY SPECIFICALLY HAVE AGREED TO IT.

2) Please ensure that the paper adheres to the PLOS Data Availability Policy (see http://journals.plos.org/plosmedicine/s/data-availability), which requires that all data underlying the study's findings be provided in a repository or as Supporting Information. For data residing with a third party, authors are required to provide instructions with contact information for obtaining the data. PLOS journals do not allow statements supported by "data not shown" or "unpublished results." For such statements, authors must provide supporting data or cite public sources that include it.

3) Please review your reference list to ensure that it is complete and correct. If you have cited papers that have been retracted, please include the rationale for doing so in the manuscript text, or remove these references and replace them with relevant current references. Any changes to the reference list should be mentioned in the rebuttal letter that accompanies your revised manuscript.

# Author comment: Author response to Decision Letter 3

**DOI:** 10.1371/journal.pmed.1005132.r005

28 Jan 2026

Attachment Submitted filename: R2_ Authors Response.docx

# Editor report: Decision Letter 3

**DOI:** 10.1371/journal.pmed.1005132.r006

**Authors:** Alexandra Tosun

9 Feb 2026

Dear Dr. Andualem,

Thank you very much for re-submitting your manuscript "A Multilevel Onsite Training and Mentorship Model to Accelerate Early Childhood Cancer Diagnosis in Northwest Ethiopia" (PMEDICINE-D-25-02106R3) for review by PLOS Medicine.

Thank you for your detailed response to the reviewers' and editor’s comments. There are several remaining editorial comments that need to be addressed before we can accept the manuscript for publication. When submitting your revised paper, please once again include a detailed point-by-point response to the editorial comments. Please revise the paper accordingly, and submit the final revision by February 16 2026 . The remaining issues that need to be addressed are listed at the end of this email.

In revising the manuscript for further consideration here, please ensure you address the specific points made by each reviewer and the editors. In your rebuttal letter you should indicate your response to the reviewers' and editors' comments and the changes you have made in the manuscript. Please submit a clean version of the paper as the main article file. A version with changes marked must also be uploaded as a marked up manuscript file. Please also check the guidelines for revised papers at http://journals.plos.org/plosmedicine/s/revising-your-manuscript for any that apply to your paper.

In addition to these revisions, you may need to complete some formatting changes, which you will receive in a follow up email. A member of our team will be in touch with a set of requests shortly. If you do not receive a separate email within a few days, please assume that checks have been completed, and no additional changes are required.

A reminder that when your manuscript is accepted, an uncorrected proof of your manuscript will be published online ahead of the final version, unless you've already opted out via the online submission form. If, for any reason, you do not want an earlier version of your manuscript published online or are unsure if you have already indicated as such, please let the journal staff know immediately at plosmedicine@plos.org.

If you have any questions in the meantime, please contact me (atosun@plos.org) or the journal staff on plosmedicine@plos.org.

We look forward to receiving the revised manuscript.

Sincerely,

Alexandra Tosun, PhD

Senior Editor

PLOS Medicine

plosmedicine.org

***Please note while forming your response, if your article is accepted, you may have the opportunity to make the peer review history publicly available. The record will include editor decision letters (with reviews) and your responses to reviewer comments. If eligible, we will contact you to opt in or out.***

------------------------------------------------------------

Requests from Editors:

Unfortunately, it is often unclear whether responses to our editorial requests have been implemented in the manuscript. Several requests have not been addressed properly. Please thoroughly revise the manuscript and take into account the comments below, as well as those from the previous decision letter. A careful revision will prevent the need for further revisions. Also, please make sure that you only upload the latest versions of files to the file inventory.

GENERAL

* We suggest changing the title to: “A Multilevel Onsite Training and Mentorship Model to Accelerate Early Childhood Cancer Diagnosis in Northwest Ethiopia: A Quasi-experimental mixed method study”

* Please ensure that all abbreviations are defined at first use throughout the text. This also includes statistical abbreviations.

* Please ensure that tables and figures, including those in supplementary files, are appropriately referenced in the main text.

* Please define all abbreviations used in each figure or table in the corresponding figure description or below the table.

* Please remove descriptions such as “paediatric oncology patients” or “cancer patients” and use patient-centered language instead. Please note that patient-centered language is constructed with the use of post-modified nouns putting the person first in the sentence structure (e.g. “patients with cancer”).

* The Data Availability Statement (DAS) requires revision. In the manuscript and the online submission form you have stated that “All relevant data is available within the manuscript and Supporting Information files”. However, in your last rebuttal you have stated that “the data are owned by a third party but are freely available upon request” and that you “have provided the owner of the dataset and the appropriate contact information for data requests”. Unfortunately, we cannot find an updated statement with the appropriate contact information. Please revise your Data Availability Statement to ensure it is accurate and complies with our requirements.

* Thank you for providing your COREQ checklist. Please replace the text excerpts with paragraph numbers per section (e.g. "Methods, paragraph 1").

* Please note that you stated that you added a completed CROSS checklist as S2 file and added a statement in the Methods section mentioning the CROSS checklist. However, we can neither find the completed checklist nor the statement.

* “Where data were available, we have added a comparison of key characteristics between survey respondents and nonrespondents in the Methods/Results section to assess potential nonresponse bias. Where such comparisons were not feasible, this has been clearly acknowledged as a limitation.” – We cannot seems to find any data on nonrespondents in the manuscript. Please comment.

* “The survey response rate has now been added to the Abstract to improve transparency and reporting completeness.” – It seems that this has not been included in the Abstract.

* “A complete copy of the survey instrument has been added to the Supplementary Files for reference and transparency.” – It seems this file has not been uploaded.

ABSTRACT

Please note that the comments and questions we raised in our previous decision letter were added because these details are missing from the Abstract. Although we appreciate your detailed responses, it appears that you have not included the relevant information in the Abstract. Please carefully revise the Abstract, taking into account the comments below and from the previous decision letter.

* Abstract Background: The final sentence should clearly state the study question.

* Please note that a very brief definition of the three tiers of care needs to be in the Abstract.

* “A six-month mentorship program combined monthly onsite visits with remote supervision.” – This statement still lacks detail. Please describe who was in the mentor group and who the mentees were and what remote supervision entails.

* “Median practice proficiency increased to 100 at primary level and to 93.8 at secondary level.” – Why did you remove the percentage? What were the pre-intervention levels? It’s impossible to judge the impact of the intervention without knowing the pre-intervention levels (as done in the prior sentence).

AUTHOR SUMMARY

* “Median provider knowledge scores increased to 90.9, and practice proficiency increased to 100 at primary level and 93.8 at secondary level.” – Similar to our comment above, we ask you to include both pre- and post-intervention values.

INTRODUCTION

* Based on the track changes and a comparison of the current and prior manuscript versions, it doesn't appear that you have streamlined the introduction. The introduction is still repetitive in some parts and needs to be streamlined. Please revise it accordingly.

METHODS AND RESULTS

* Figure 1: In the rebuttal, you indicated that you translated the figure text. However, the translation appears to be missing.

* While Figure 2 depicts the type of delays, it does not contain any information on the intervention timeline, the assessment types (i.e., main outcome measure) and when these assessments took place. Please re-consider adjusting the figure.

* """Two analysts independently coded transcripts,” – please provide the initials. Response to the editors: Thank you very much for the comments. We have included it in the abstract section.""" – Please note that we did not ask you to include this detail in the abstract. Rather, we asked you to mention the initials of the two analysts so that they can be identified in the author/acknowledgment list.

* Table 1: The definition for the asterisk (Other*), ‘BSc’, and ‘MSc/MPH’ is missing below the table. Please add.

* Table 2: The definition for ‘Certificate’ and ‘first degree’ is missing below the table. Please add.

* Figure 3: Please add a unit for the y-axis in the graph.

* “Figure 3: Please ensure to provide the relevant data behind the graphs in a supplementary table.” - You have stated that you have included the necessary data, but you don’t cite any supplementary table. Where can readers find the values behind the graph?

* Table 3: When reporting a median, please include the interquartile range.

* “Seven hundred forty-seven (70 %) of participants reported that joint oncology case conferences…” – please ensure to provide numerator and denominator. Please revise throughout the entire manuscript.

DISCUSSION

* Please remove all subheadings from the discussion (Limitations, Conclusions).

# Author comment: Author response to Decision Letter 4

**DOI:** 10.1371/journal.pmed.1005132.r007

25 Feb 2026

Attachment Submitted filename: Authors response to editors_R3.docx

# Editor report: Decision Letter 4

**DOI:** 10.1371/journal.pmed.1005132.r008

**Authors:** Alexandra Tosun

26 Feb 2026

Dear Dr. Andualem,

Thank you very much for re-submitting your manuscript "A Multilevel Onsite Training and Mentorship Model to Accelerate Early Childhood Cancer Diagnosis in Northwest Ethiopia: Quasi-experimental mixed method study" (PMEDICINE-D-25-02106R4) for review by PLOS Medicine.

Thank you for addressing our editorial requests again. Unfortunately, we still feel that some of our requests have not been adequately addressed. Please note that a rebuttal should indicate where you have implemented changes, for example, by providing line numbers.

We are unsure where the miscommunication arises from. However, to avoid further delays, we strongly recommend having a professional scientific copy editor check the manuscript. We suggest showing them the last three decision letters (R2, R3, and R4) and working with them through the manuscript. We want to swiftly move the manuscript towards acceptance, but cannot do so unless it complies with our journal requirements.

If you have any questions in the meantime, please contact me at atosun@plos.org.

We look forward to receiving the revised manuscript by Mar 05 2026.

Sincerely,

Alexandra Tosun, PhD

Senior Editor

PLOS Medicine

plosmedicine.org

***Please note while forming your response, if your article is accepted, you may have the opportunity to make the peer review history publicly available. The record will include editor decision letters (with reviews) and your responses to reviewer comments. If eligible, we will contact you to opt in or out.***

------------------------------------------------------------

Requests from Editors:

* TITLE: Please change the title to (use the exact wording): “Multilevel onsite training and mentorship model to accelerate early childhood cancer diagnosis in Northwest Ethiopia: A quasi-experimental mixed method study”

* CHECKLISTS – please address all comments carefully:

1) Please include the following statement in the Methods section: “This study is reported as

per the Consolidated criteria for reporting qualitative research (COREQ): a 32-item

checklist for interviews and focus groups (S2 Checklist).”

2) Additionally, please upload the complete CROSS checklist as supporting information file. Complete the CROSS checklist with paragraph numbers per section (e.g. "Methods, paragraph 1") and add the following statement in the Methods section: “This survey-based part of our study is reported as per A Consensus-Based Checklist for Reporting of Survey Studies (CROSS) guideline (S1 Checklist)." – If you don’t think the CROSS checklist is needed for your study, please state so.

* ABSTRACT: We have revised the abstract to include the details we previously requested, which you still have not included. Please see the attached file.

* ABSTRACT: Please note that any numerical results reported in the abstract must include uncertainty ranges, such as 95% confidence intervals. We have highlighted all instances requiring these details in green. If you are unsure about this, we suggest contacting a statistician.

* TABLE 3: Please note that "27 ± 49" cannot be the median plus the interquartile range (IQR). Please consult a statistician.

* FIGURE 2: Please provide the data behind the graphs in a supplementary tables. S3 file contains the survey and the data behind the graphs is still missing. We will not be able to publish the manuscript without the data.

Attachment Submitted filename: Abstract_PMEDICINE-D-25-02106R4.docx

# Author comment: Author response to Decision Letter 5

**DOI:** 10.1371/journal.pmed.1005132.r009

9 May 2026

Attachment Submitted filename: Response to Editors_ R6.docx

# Editor report: Decision Letter 5

**DOI:** 10.1371/journal.pmed.1005132.r010

**Authors:** Katarzyna Starowicz

22 May 2026

Dear Dr Andualem,

On behalf of my colleagues and the Academic Editor, James K Tumwine, I am pleased to inform you that we have agreed to publish your manuscript "Multilevel Onsite Training and Mentorship Model to Accelerate Early Childhood Cancer Diagnosis in Northwest Ethiopia: A Quasi-experimental mixed method study" (PMEDICINE-D-25-02106R5) in PLOS Medicine.

Before your manuscript can be formally accepted you will need to complete some formatting changes, which you will receive in a follow up email. Please be aware that it may take several days for you to receive this email; during this time no action is required by you. Once you have received these formatting requests, please note that your manuscript will not be scheduled for publication until you have made the required changes.

In the meantime, please log into Editorial Manager at http://www.editorialmanager.com/pmedicine/, click the "Update My Information" link at the top of the page, and update your user information to ensure an efficient production process.

PRESS

We frequently collaborate with press offices. If your institution or institutions have a press office, please notify them about your upcoming paper at this point, to enable them to help maximise its impact. If the press office is planning to promote your findings, we would be grateful if they could coordinate with medicinepress@plos.org. If you have not yet opted out of the early version process, we ask that you notify us immediately of any press plans so that we may do so on your behalf.

We also ask that you take this opportunity to read our Embargo Policy regarding the discussion, promotion and media coverage of work that is yet to be published by PLOS. As your manuscript is not yet published, it is bound by the conditions of our Embargo Policy. Please be aware that this policy is in place both to ensure that any press coverage of your article is fully substantiated and to provide a direct link between such coverage and the published work. For full details of our Embargo Policy, please visit http://www.plos.org/about/media-inquiries/embargo-policy/.

To enhance the reproducibility of your results, we recommend that you deposit your laboratory protocols in protocols.io, where a protocol can be assigned its own identifier (DOI) such that it can be cited independently in the future. Additionally, PLOS ONE offers an option to publish peer-reviewed clinical study protocols. Read more information on sharing protocols at https://plos.org/protocols?utm_medium=editorial-email&utm_source=authorletters&utm_campaign=protocols

Thank you again for submitting to PLOS Medicine. We look forward to publishing your paper.

Sincerely,

Katarzyna Starowicz, PhD

Senior Editor

PLOS Medicine
